# PFKM inhibits doxorubicin-induced cardiotoxicity by enhancing oxidative phosphorylation and glycolysis

**DOI:** 10.1038/s41598-022-15743-0

**Published:** 2022-07-08

**Authors:** Min Zhou, Xiao Sun, Chunli Wang, Fengdan Wang, Chuibi Fang, Zhenlei Hu

**Affiliations:** Department of Cardiovascular Surgery, Affiliated 9th People’s Hospital, School of Medicine, Shanghai Jiaotong University, Shanghai, 201900 China

**Keywords:** Heart failure, Glycobiology

## Abstract

Heart failure (HF) is a global pandemic which affects about 26 million people. PFKM (Phosphofructokinase, Muscle), catalyzing the phosphorylation of fructose-6-phosphate, plays a very important role in cardiovascular diseases. However, the effect of PFKM in glycolysis and HF remains to be elucidated. H9c2 rat cardiomyocyte cells were treated with doxorubicin (DOX) to establish injury models, and the cell viability, apoptosis and glycolysis were measured. Quantitative reverse transcription-polymerase chain reaction (RT-PCR) and immunoblotting were used for gene expression. DOX treatment significantly inhibited PFKM expression in H9c2 cells. Overexpression of PFKM inhibited DOX-induced cell apoptosis and DOX-decreased glycolysis and oxidative phosphorylation (OXPHOS), while silencing PFKM promoted cell apoptosis and inhibited glycolysis and OXPHOS in H9c2 cells. Moreover, PFKM regulated DOX-mediated cell viability and apoptosis through glycolysis pathway. Mechanism study showed that histone deacetylase 1 (HDAC1) inhibited H3K27ac-induced transcription of PFKM in DOX-treated cells and regulated glycolysis. PFKM could inhibit DOX-induced cardiotoxicity by enhancing OXPHOS and glycolysis, which might benefit us in developing novel therapeutics for prevention or treatment of HF.

## Introduction

Heart failure (HF) is a clinical syndrome caused by defects in myocardium resulting in impairment of ventricular filling or the ejection of blood^1^. HF symptoms include breathlessness, ankle swelling, and fatigue, accompanied by pulmonary rales, peripheral oedema, etc.^2^. HF is a global pandemic and its prevalence is still increasing^3^. The major HF risk factors heart disease, cardiopulmonary disease, etc.^4^. Evaluation factors for HF include physical examination, blood tests, levels of serum creatinine and glucose, liver function tests etc.^1^. Measurement of plasma concentrations of brain natriuretic peptide is a mainstay for the diagnosis of HF^5^. The burden of HF is huge, study show that estimated mean cost of HF was $11 552 in 2014 in USA^6^. More importantly, HF is still increasing in prevalence^3^. Therefore, a better understanding of the pathogenesis of HF will benefit us in the prevention and treatment of HF.

Hyperglycemia by impairing glucose metabolism is an emerging risk factor for cancer and cardiovascular disease. Hyperglycemia reduces ipilimumab-related anticancer functions and enhances its cardiotoxicity through mechanisms mediated by MyD88 and NLRP3 signaling^7^, suggesting that targeting the MyD88/NLRP3 signaling may be beneficial in patients with cancer and cardiovascular diseases in response to ipilimumab-induced anticancer effects and cardiotoxicity. Glucose can be metabolized by glycolysis to lactate. PFKM (Phosphofructokinase, Muscle) regulates glycolysis via catalyzing the phosphorylation of fructose-6-phosphate^8^. PFKM has been shown to be involved in various pathological processes. For example, Gao et al. have reported that S-nitrosylation at Cys351 of PFKM promoted cell proliferation, and increased tumor growth and metastasis of ovarian cancer^9^. A study identified PFKM as a breast cancer gene^10^. Another study indicated that celastrol directly inhibits PFKM to induce weight loss^11^. PFKM also plays a very important role in cardiovascular diseases. For example, PFKM mutation causes myopathy^8^. PFK deficiency results in a severe cardiac and hematological disorder^12^. However, the exact role of PFKM in cardiotoxicity remains elusive.

Glycolysis and oxidative phosphorylation (OXPHOS) produce energy for cells^13^. OXPHOS is the process by which ATP synthesis is coupled to the movement of electrons through the mitochondrial electron transport chain and the associated consumption of oxygen^14^. Glycolysis converts glucose to lactate and provides ATP under anaerobic conditions^15^. Dysfunction of OXPHOS or glycolysis has been associated with a variety of diseases. For instance, it has been reported that defects in OXPHOS in insulin-sensitive tissues contribute to type 2 diabetes^16^. Defect of OXPHOS system has been linked to neurodegeneration including Alzheimer disease, Huntington disease, etc.^17^. An adipocyte-specific defect in OXPHOS increases systemic energy expenditure^18^. OXPHOS also regulates cardiovascular diseases. For example, coronary artery disease subjects showed suppressed function of complexes I, II and III^19^. Glycolysis also plays a role in HF. Increased glycolysis was found in failing hearts^20^. HF animals showed a remarkable increase in glycolysis^21^. Despite the advance of studies on HF, PFKM, and OXPHOS/glycolysis, the exact role of PFKM in OXPHOS/glycolysis and how it does affect HF are still unclear and remain to be elucidated.

## Results

### DOX treatment inhibited PFKM expression in H9c2 cells

**Figure 1:**
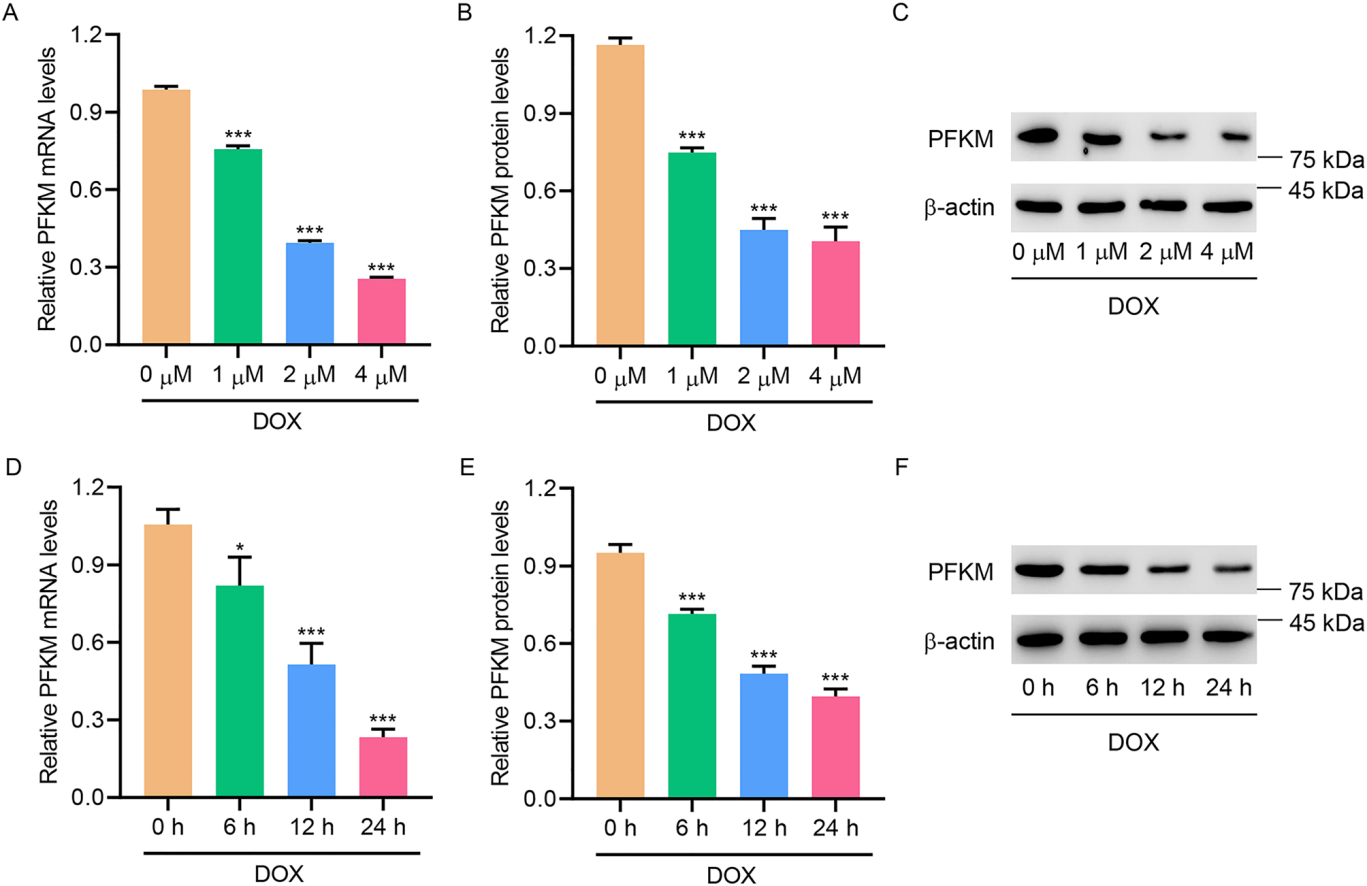
DOX treatment inhibited PFKM expression in H9c2 cells. (**A**–**C**) PFKM expression in DOX-treated H9c2 cells. (**D**–**F**) PFKM expression in DOX-treated H9c2 cells for different time points. *P < 0.05, ***P < 0.001 vs 0 μM or 0 h. (see Supplementary Fig. S1–S4)

To study the role of PFKM in cardiac injury, DOX treatment was used to induce in vitro cardiac injury. DOX treatment not only dose-dependently decreased the expression of PFKM (Fig. 1A–C), but also time-dependently suppressed the expression of PFKM (Fig. 1D–F), suggesting that DOX inhibited PFKM expression in H9c2 cells.

### PFKM upregulation abolished DOX-induced cell apoptosis and DOX-suppressed OXPHOS and glycolysis in H9c2 cells

**Figure 2:**
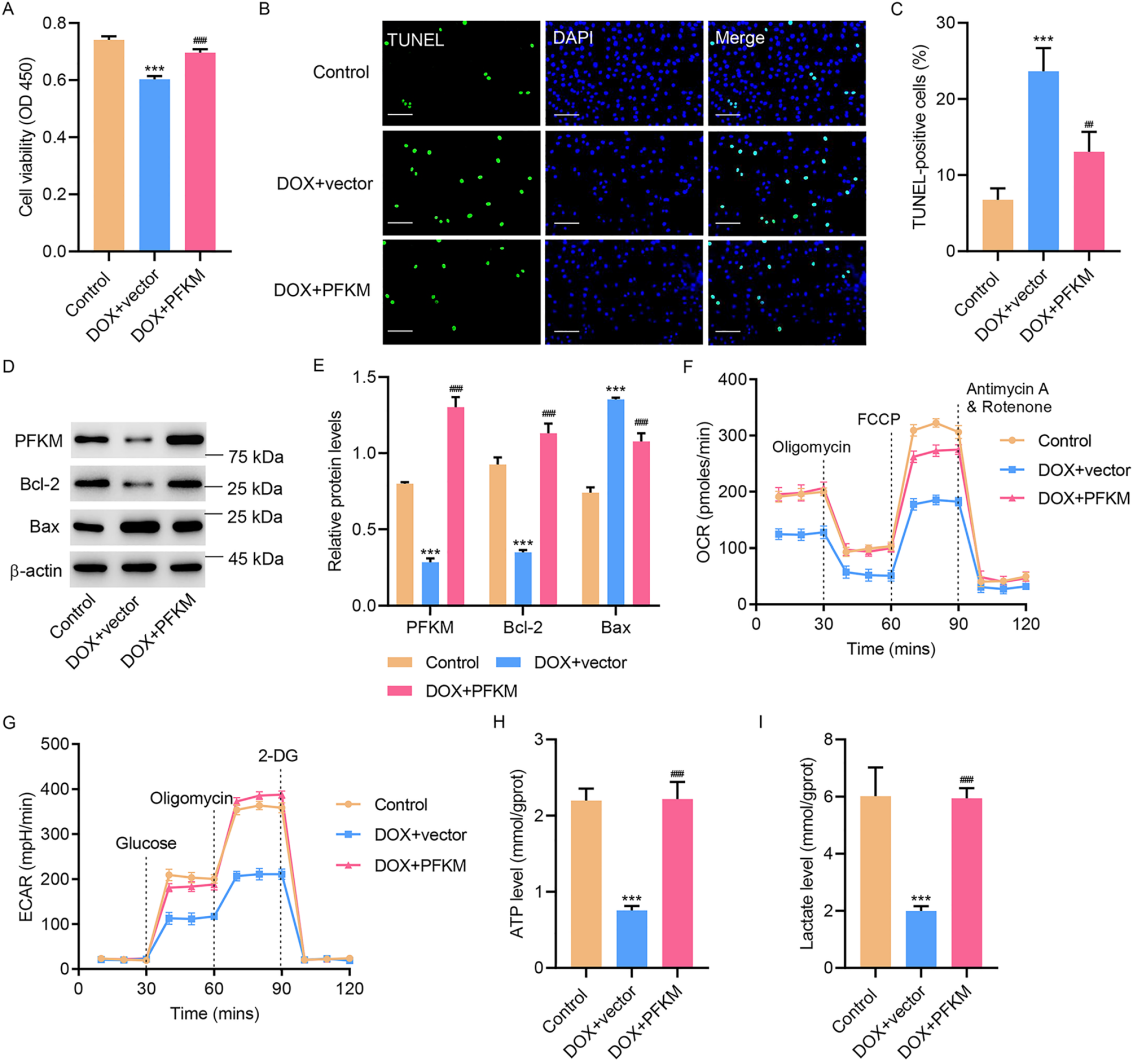
PFKM upregulation inhibited DOX-induced cell apoptosis and DOX-decreased OXPHOS and glycolysis. PFKM-overexpressing H9c2 cells were treated with DOX for 24 h, and (**A**) cell viability, (**B**, **C**) TUNEL staining, (**D**, **E**) expression of PFKM, Bcl-2 and Bax, (**F**) OCR, (**G**) ECAR, (**H**) ATP and (**I**) lactate level were measured. Scale bar: 100 μm. ***P < 0.001 vs Control; ^##^P < 0.01, ^###^P < 0.001 vs DOX + vector. (see Supplementary Fig. S5–S8)

To further investigate the role of PFKM, PFKM was overexpressed. PFKM overexpression significantly increased DOX-inhibited cell viability (Fig. 2A), decreased DOX-promoted apoptosis (Fig. 2B,C), and reversed DOX-decreased PFKM, Bcl-2, and DOX-increased Bax (Fig. 2D,E). Overexpression of PFKM also abolished DOX-inhibited OCR and ECAR (Fig. 2F,G), and reversed DOX-decreased levels of ATP (Fig. 2H) and lactate levels (Fig. 2I). The results show that overexpressing PFKM abolished DOX-induced cell apoptosis and DOX-suppressed OXPHOS and glycolysis.

### PFKM downregulation promoted cell apoptosis and inhibited OXPHOS and glycolysis in H9c2 cells

**Figure 3:**
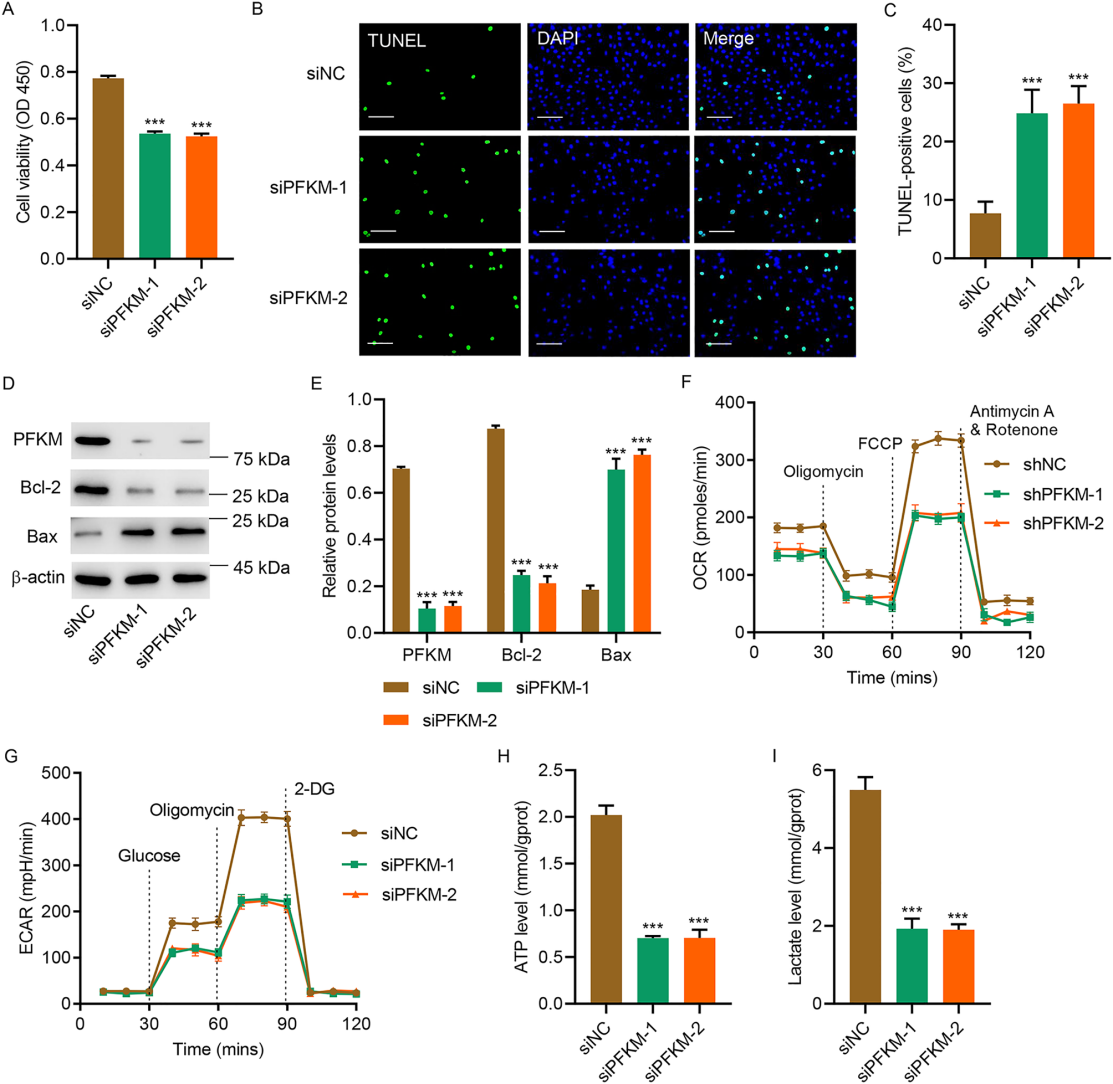
PFKM downregulation promoted cell apoptosis and inhibited OXPHOS and glycolysis. H9c2 cells were transfected with PFKM siRNA or siNC, and (**A**) cell viability, (**B**, **C**) TUNEL staining, (**D**, **E**) expression of PFKM, Bcl-2 and Bax, (**F**) OCR, (**G**) ECAR, (**H**) ATP and (**I**) lactate level were measured. Scale bar: 100 μm. ***P < 0.001 vs siNC. (see Supplementary Fig. S9–S12, more information for Supplementary Fig. S12 can be found in Supplementary Information 1.)

Next, PFKM was silenced to further study its role. Silencing PFKM significantly inhibited cell viability (Fig. 3A), promoted apoptosis (Fig. 3B,C), and inhibited PFKM, Bcl-2, but increased Bax (Fig. 3D,E). Silencing PFKM also suppressed OCR and ECAR (Fig. 3F,G), and decreased levels of ATP (Fig. 3H) and lactate (Fig. 3I). The results demonstrate that PFKM downregulation promoted cell apoptosis and inhibited OXPHOS and glycolysis in H9c2 cells.

### PFKM regulated DOX-mediated cell viability and apoptosis via glycolysis

**Figure 4:**
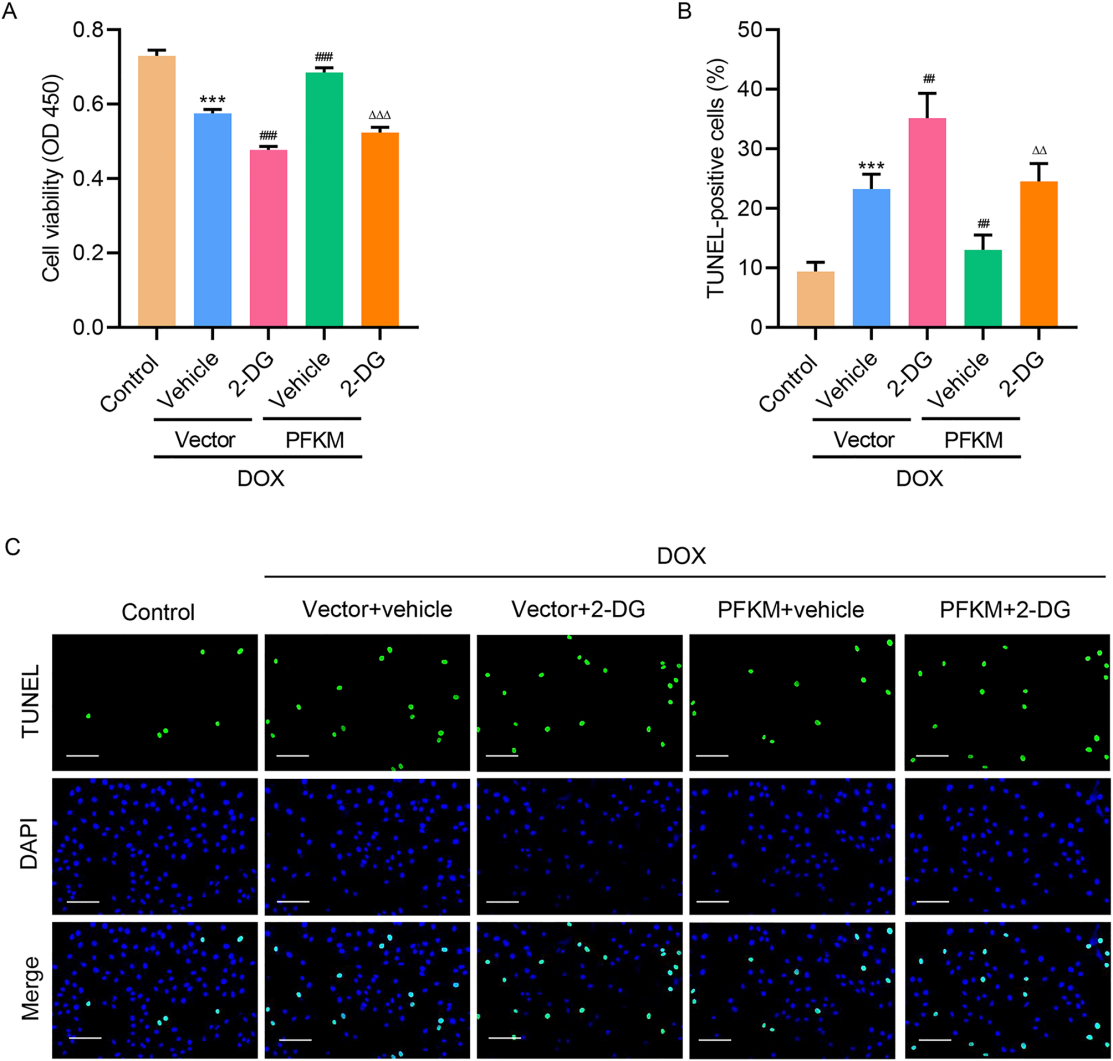
PFKM regulates DOX-mediated cell growth and apoptosis via the glycolysis pathway. PFKM-overexpressing H9c2 cells were treated with DOX with/without 2-DG for 24 h, and (**A**) cell viability and (**B**, **C**) TUNEL staining was measured. Scale bar: 100 μm. ***P < 0.001 vs Control; ^##^P < 0.01, ^###^P < 0.001 vs DOX + vector + vehicle; ^ΔΔ^P < 0.01, ^ΔΔΔ^P < 0.001 vs DOX + PFKM + vehicle.

To find out how PFKM regulates cell viability, glycolysis inhibitor, 2-DG, was introduced. Results showed that inhibition of glycolysis not only significantly promoted DOX-suppressed cell viability, but also abolished PFKM-overexpression-increased cell viability (Fig. 4A). TUNEL staining results showed that inhibition of glycolysis not only significantly increased DOX-increased cell apoptosis, but also abolished PFKM-overexpression-decreased cell apoptosis (Fig. 4B,C). The findings suggest that PFKM regulates DOX-mediated growth and apoptosis via the glycolysis pathway.

### HDAC1 inhibited H3K27ac-induced transcription of PFKM in DOX-induced H9c2 cells

**Figure 5:**
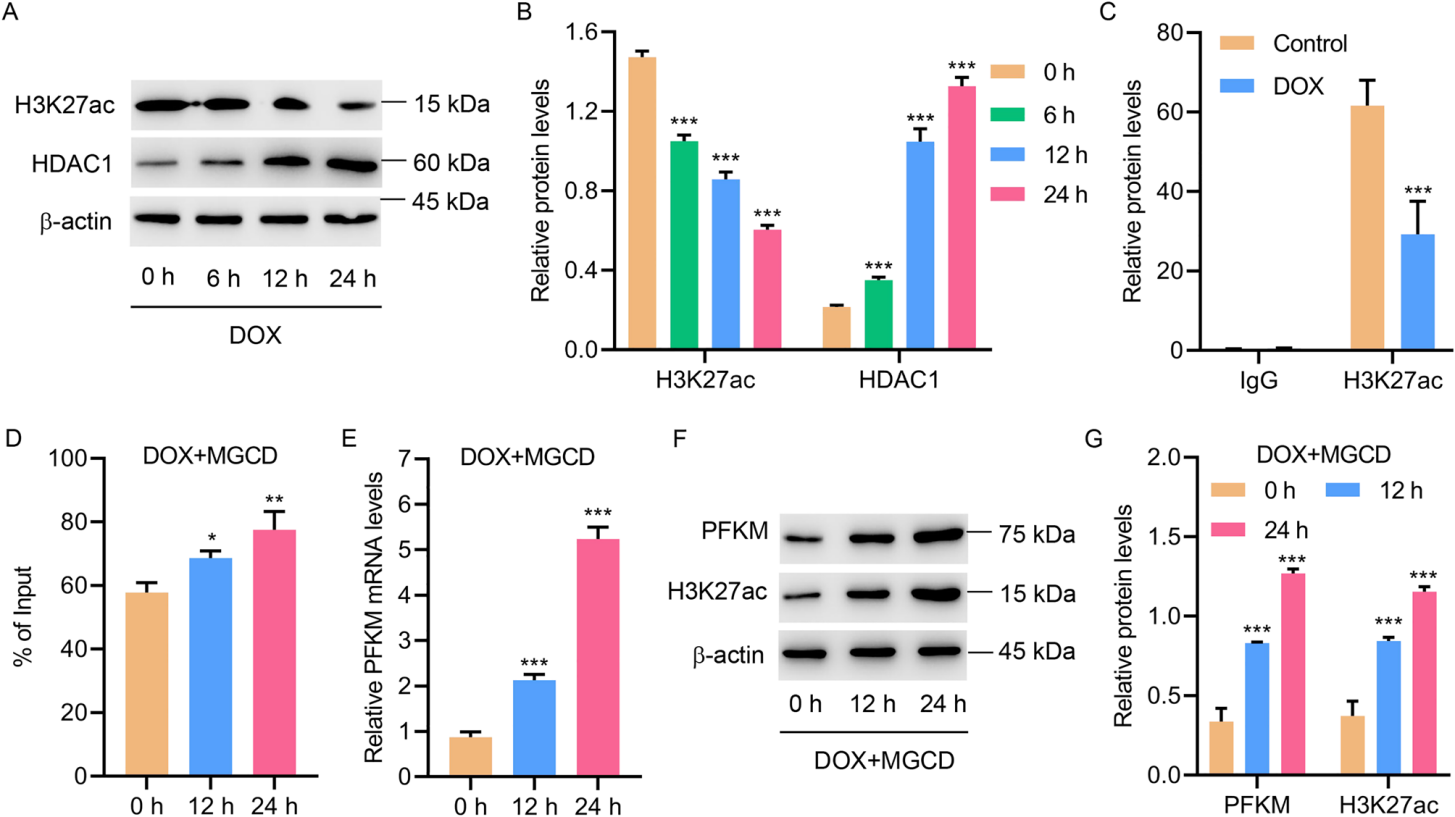
HDAC1 inhibited H3K27ac-induced transcription of PFKM in DOX-induced H9c2 cells. (**A**, **B**) H3K27ac and HDAC1 levels in DOX-treated H9c2 cells. (**C**) ChIP assay of H3K27ac on the PFKM promoter in DOX-treated H9c2 cells. (**D**) ChIP assay of H3K27ac on the PFKM promoter and (**E**–**G**) expression of PFKM and H3K27ac in H9c2 cells treated with 2 μM DOX and 0.1 μM MGCD for different time points. *P < 0.05, **P < 0.01, ***P < 0.001 vs 0 h or control. (see Supplementary Fig. S13–S18)

To figure out the mechanism by which PFKM is regulated, levels of H3K27ac and HDAC1 in DOX-treated H9c2 cells were measured. Results showed that DOX suppressed H3K27ac in a time-dependent manner, but increased the expression of HDAC1 (Fig. 5A,B). ChIP assay revealed that DOX treatment significantly suppressed the interaction between H3K27ac and the PFKM promoter (Fig. 5C). HDAC inhibitor, mocetinostat (MGCD), significantly promoted the interaction between H3K27ac and the PFKM promoter (Fig. 5D) and increased PKFM and H3K27ac expression in DOX-treated H9c2 cells (Fig. 5E–G). Together, these results suggest that HDAC1 inhibited H3K27ac-induced transcription of PFKM in DOX-induced H9c2 cells.

### HDAC1-induced PFKM transcriptional repression regulated DOX-mediated OXPHOS and glycolysis

**Figure 6:**
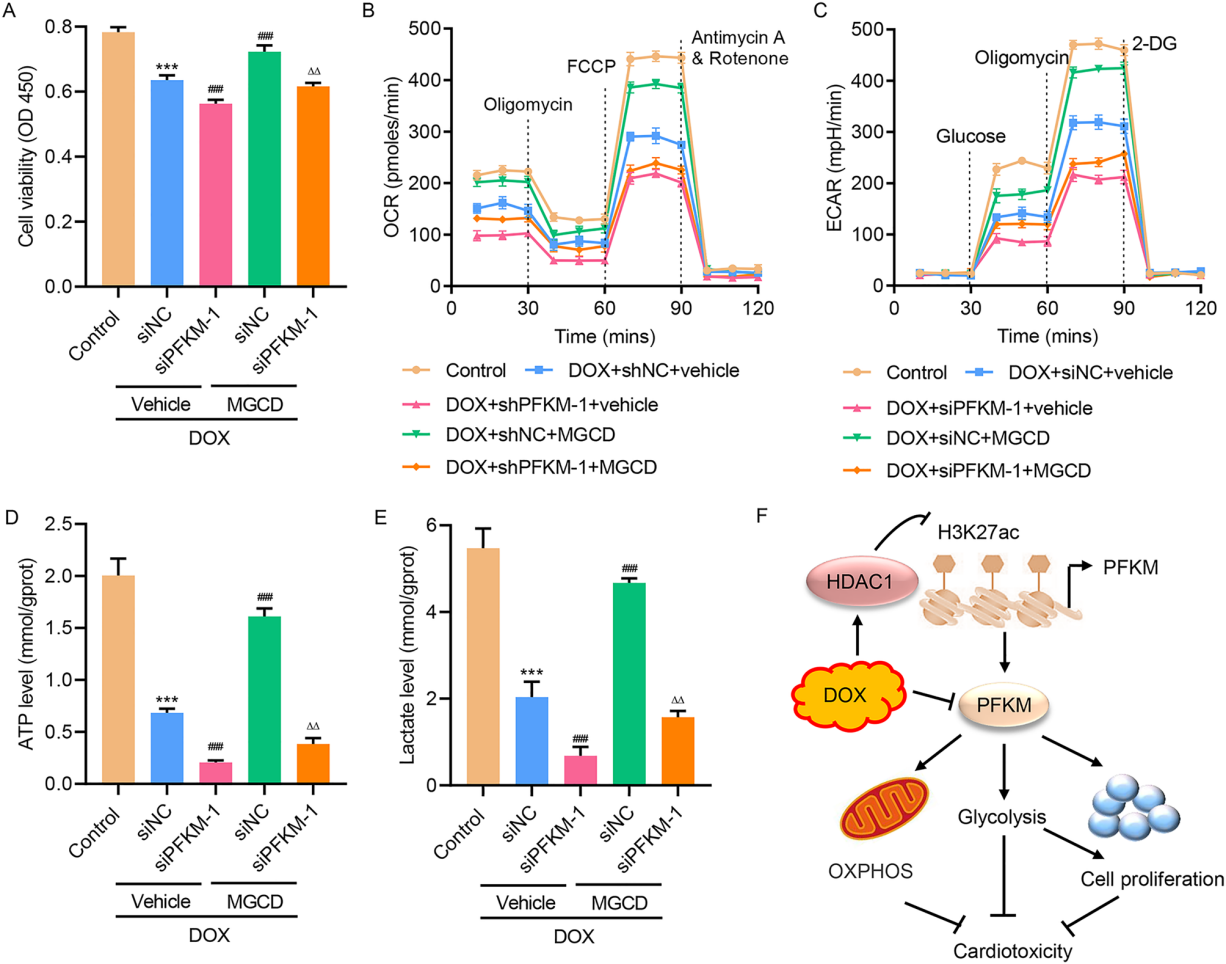
HDAC1-induced PFKM transcriptional repression regulated DOX-mediated OXPHOS and glycolysis in H9c2 cells. H9c2 cells were transfected with PFKM siRNA or nonspecific siRNA (siNC) and treated with 2 μM DOX in the absence or presence of 0.1 μM MGCD for 24 h, and (**A**) cell viability, (**B**) OCR and (**C**) ECAR, (**D**) ATP and (**E**) lactate level were measured. (**F**) Schematic representation of the regulation of DOX-induced cardiotoxicity via OXPHOS and glycolysis through HDAC1-induced PFKM transcriptional repression. ***P < 0.001 vs Control; ^###^P < 0.001 vs DOX + siNC + vehicle; ^ΔΔ^P < 0.01 vs DOX + siPFKM-1 + vehicle.

To further study the role of HDAC1, MGCD and DOX were used to treat H9c2 cells transfected with PFKM small interfering RNA (siRNA) or nonspecific siRNA (siNC). Results showed that MGCD treatment abolished DOX-suppressed cell viability (Fig. 6A), and ameliorated DOX-inhibited OCR and ECAR (Fig. 6B,C). MGCD treatment also reversed DOX-decreased levels of ATP (Fig. 6D) and lactate (Fig. 6E), suggesting that HDAC1-induced PFKM transcriptional repression regulated DOX-mediated OXPHOS and glycolysis in H9c2 cells.

## Discussion

We revealed that DOX treatment significantly inhibited PFKM expression in H9c2 cells. Overexpressing of PFKM inhibited DOX-induced cell apoptosis and DOX-decreased glycolysis, while silencing PFKM promoted cell apoptosis and inhibited OXPHOS and glycolysis in H9c2 cells. Moreover, PFKM regulated DOX-mediated cell growth and apoptosis via glycolysis pathway. Data also supports that the expression of PFKM was suppressed by HDAC1 through regulating H3K27 acetylation. For the first time, we show that HDAC1-mediated PFKM down-regulation promoted cell apoptosis and inhibited OXPHOS in H9c2 cells through regulating glycolysis (Fig. 6F), which may provide novel directions for new drug development.

Glycolysis regulated energy metabolism^22^. Glucose is converted into pyruvate, NADH, and ATP by glycolysis^23^. Glycolysis involves in many biological and pathological processes. For example, glycolysis promotes tumor growth^24^. Preclinical studies demonstrate that some small molecules such as 3-bromopyruvate suppresses cancer via targeting glycolysis^25^. Another study showed that Smad4 depletion in podocytes protects mice from glomerulosclerosis^26^. Inhibition of aerobic glycolysis causes depression of cardiac excitability and can lead to Ca^2+^ alternant in cardiac tissue^27^. Increased glycolysis is the earliest energy metabolic change during heart failure with preserved ejection fraction^28^. Other studies have demonstrated that glycolysis affects sarcoplasmic reticulum (SR) function and SR Ca^2+^ release not only through generation of ATP but also through direct interactions of glycolytic intermediates and products with the Ca^2+^ release channel itself^27^. Moreover, hyperglycemia enhances ipilimumab-induced cardiotoxicity through mechanisms mediated by MyD88 and NLRP3 signaling^7^, suggesting that targeting the MyD88/NLRP3 signaling may inhibit ipilimumab-induced cardiotoxicity in patients with cardiovascular diseases. In this study, we demonstrated that suppressing glycolysis remarkably ameliorated the effect of PFKM on DOX-mediated cell growth and apoptosis. These results reveal a very important role of glycolysis in regulating DOX-mediated cell growth and apoptosis and improve our knowledge of the role of glucose homeostasis in the reduction of doxorubicin cardiotoxicity.

PFK catalyzes the rate-limiting phosphorylation of fructose-6-phosphate and sustains a high rate of glycolysis^8^. It has 3 isoforms: platelet (PFKP), muscle (PFKM), and liver (PFKL)^29^. PFKM gene has 24 exons^30^. PFK deficiency belongs to glycogen storage disease characterized by weakness with spasms and cramping on exercise^30^. Ristow et al. have reported that deficiency of PFKM results in insulin resistance, contributing to diabetes^31^. Studies also indicate that PFKM plays a very important role in cardiovascular diseases. For instance, Garcia et al. indicated that PFK deficiency causes a cardiac and hematological disorder^12^. Two-month-old PFKM knockout mice developed cardiac hypertrophy and evident cardiomegaly with age^12^. Preclinical studies correlate high levels of IL-1β to a greater risk of cardiovascular diseases; the underlying mechanism of cardiotoxicity involves the dysfunction of mitochondrial metabolism^32^. Therefore, pharmacological inhibition of IL-1β could be a promising approach for the treatment of cardiovascular diseases. In this study, PFKM downregulation also inhibited OXPHOS in H9c2 cells. However, the role of IL-1β in PFKM-induced glucose and mitochondrial metabolism in DOX-treated H9c2 cells need further investigation. Our findings indicate a very important role of PFKM in regulating the proliferation of cardiomyoblasts and cardiotoxicity and improve our knowledge of FPKM in the pathogenesis of HF.

Protein acetylation is the process that the acetyl group is transferred to a polypeptide chain^33^. Acetylation alters protein function^33^. Protein acetylation plays a very important role in diverse physiological processes^34,35^. H3K27ac involves in the higher activation of transcription^36^. It is elevated in mammary cancer and administration of H3K27ac inhibitor repressed tumor formation^37^. Felice et al. demonstrated that hypoacetylation of H3K27 involves in intestinal inflammation^38^. H3K27ac also involves in cardiovascular diseases. For example, a study indicated that H3K27ac acetylation status regulates phenotypic response in HF^39^. Papait et al. reported that H3K27ac was decreased in mice with transverse aortic constriction^40^. Our findings suggested that HDAC1 significantly decreased the level of H3K27ac to suppress the transcription of PFKM and regulate OXPHOS and glycolysis. These findings indicated the significance of HDAC1/H3K27ac/PFKM axis in cardiotoxicity and HF, which may benefit the study of HF and cardiovascular diseases. Keep in mind that only in vitro cell experiments were used in this study. Future studies with animals or even clinical samples will definitely supply more meaningful data. Nevertheless, our study revealed new roles of PFKM and glycolysis in HF.

In conclusion, PFKM could inhibit doxorubicin-induced cardiotoxicity by enhancing OXPHOS and glycolysis. The findings demonstrate significance of PFKM and glycolysis which might benefit us in developing novel therapeutics for prevention or treatment of HF.

## Methods

### Cell culture

The H9c2 cells, purchased from ATCC (Manassas, VA), were cultured in Dulbecco's modified eagle medium (DMEM) with 10% Fetal bovine serum (FBS) at 37 °C. Cardiac injury model was induced by providing different concentrations of DOX (Chroma Bio., Chengdu, China) for indicated times with/without 2-Deoxy-D-glucose (2-DG) (5 mM) or Mocetinostat (MGCD; 0.1 μM).

### Plasmids or siRNA transfection

Transfections were conducted with the Lipofectamine 3000 (Invitrogen) as per the supplier’s protocol. pcDNA3.1 plasmid was used to construct the PFKM overexpression vector. Small interfering RNAs targeting PFKM (siPFKM-1, GGAGGTATACAAGCTTCTA (sense), TAGAAGCTTGTATACCTCC (reverse) and siPFKM-2, GGCGAGTGTTTATCATCGA (sense), TCGATGATAAACACTCGCC (reverse)) and nonspecific siRNA were obtained from Shanghai GenePharma Co., Ltd. Empty vector and nonspecific siRNA were utilized as controls.

### Cell viability assay

H9c2 cells (3 × 10^3^ cell/well) were cultured in 96-well plates and incubated at 37 °C overnight. After 24 h treatment, 10 μL of the Cell Counting Kit-8 (CCK-8; Signalway Antibody LLC, College Park, MD, USA; CP002) solution was added into each well and incubated for an extra 1 h. Cell viability was subsequently determined using a microplate reader (PERLONG MEDICAL, Beijing, China; DNM-9602) at OD450nm.

### TdT-mediated dUTP nick-end labeling (TUNEL) staining

TUNEL staining was used for apoptosis^41^. Cell nucleus was stained with 4′,6-diamidino-2-phenylindole (DAPI) and observed under a fluorescence microscope.

### Extracellular flux (XF) analysis

Twenty-four hours after treatment, glycolysis and mitochondrial respiration levels were monitored by measuring extracellular acidification rate (ECAR) and oxygen consumption rate (OCR) using a Seahorse XF24 Extracellular Flux Analyzer^42^. Briely, cells digested to a density of 1 × 10^4^/well, were seeded in XF-24 culture plates (Agilent Technologies, Santa Clara, CA, USA, 100777-004), and were then placed in an incubator of 37 °C and 5% CO_2_ for 24 h. Around 1 h before detection, cells were shifted into an incubator without CO_2_, and culture medium was replaced by XF Base Medium (Agilent Technologies, Santa Clara, CA, USA, 103335-100). Subsequently, 1 μM oligomycin (ATP synthase inhibitor) was added into “A” well of Seahorse gauging plate, 1.5 μM carbonyl cyanide p-trifluoromethoxyphenylhydrazone (FCCP; uncoupler) was supplemented into “B” well and then mixture of antimycin A (complex III inhibitor; 0.5 μM) & rotenone (complex I inhibitor; 0.5 μM) was instilled into “C” well using Seahorse XF Cell Mito Stress Test Kit (Agilent Technologies, Santa Clara, CA, USA, 103015-100). Using a Seahorse XF24 Extracellular Flux Analyzer (Agilent Technologies, Santa Clara, CA, USA), cellular OCR was monitored. In addition, the cells were treated sequentially with 1 μM of glucose, 1 μM of oligomycin, and 0.5 μM of 2-DG (the glycolytic inhibitor) at time points for measurement of ECAR.

### Measurement of lactate and ATP

The cells were seeded in 96-well plates at 3.5 × 10^3^ cells per well. After overnight incubation at 37 ℃, 5% CO_2_, the complete medium was changed to fresh DMEM (50 μl/well). After 24 h, the supernatant of cells was collected by centrifugation. Then, according to the manufacturer's instructions, the lactate release was determined using Lactic Acid assay kit (Nanjing Jiancheng Bioengineering Institute, China). ATP content was measured with the ATP assay kit (Nanjing Jiancheng Bioengineering Institute, China), as per the manufacturer's protocol. In brief, cells were seeded in the 6-well plate for 12–24 h. Then cells were harvested by using 200–300 μl lysis buffer and vortexed for 1 min. The supernatant was mixed with detection solution and then analysis for ATP concentration was normalized to the corresponding total protein amounts from each sample.

### Reverse transcription-polymerase chain reaction (RT-PCR)

Total RNAs were extracted using TRIzol reagents. Genomic DNA was removed from the RNA samples using RNase-free DNase I from Fermentas Life Sciences (Thermo Fisher Scientific, MA, USA). One microgram of the total RNA (1 mg) was used to generate a single strand of cDNA using the QuantiTect Reverse Transcription Kit (Qiagen TM, DE), according to the manufacturer's instructions. Levels of interested genes were assessed by Quantitative RT-PCR using SYBR Green Master Mix (Roche, Shanghai) with following primers: PFKM: 5′-ATCACAGCCGAGGAGGCTAC-3′ (F), 5′-GGCGGCCCATCACTTCTAAC-3′ (R); β-actin: 5′-CGGTCAGGTCATCACTATC-3′ (F), 5′-CAGGGCAGTAATCTCCTTC-3′ (R). Fold change was calculated using 2^−ΔΔCt^ formula.

### Immunoblotting

Cell lysates were extracted using radioimmunoprecipitation assay buffer (JRDUN Biotechnology, Co., Ltd., Shanghai, China). Total protein concentration in each sample was measured using a Lowry protein assay kit (Bio-Rad Laboratories, Inc., Hercules, CA, USA). Equivalent quantities (25 μg) of protein were separated by 10 or 15% sodium dodecyl sulfate–polyacrylamide gel electrophoresis and transferred to nitrocellulose membranes (Sigma-Aldrich), followed by blocking in fat-free milk overnight at 4˚C. The membranes were incubated with primary antibodies, including anti-PFKM antibody (ab154804, dilution 1:1000, Abcam), anti-Bcl-2 antibody (ab182858, dilution 1:2000, Abcam), anti-Bax antibody (ab32503, dilution 1:10,000, Abcam), anti-H3K27ac antibody (ab177178, dilution 1:10,000, Abcam), anti-HDAC1 antibody (10,197–1-AP, dilution 1:8000, Proteintech), and anti-beta actin antibody (66,009 − 1-Ig, dilution 1:50,000, Proteintech) for overnight at 4 °C. The membranes were then incubated for 1 h at 37 °C with anti-horseradish peroxidase-conjugated IgG secondary antibodies (ZB-2305, ZB-2301, dilution 1:5000, ZSGB-BIO, Beijing, China). Chemiluminescence detection was conducted using Western Lightning Chemiluminescence Reagent Plus (PerkinElmer, Inc., Waltham, MA, USA) and signals were quantified by densitometry (Quantity One software, version 4.62; Bio-Rad Laboratories, Inc.).

### Chromatin immunoprecipitation (ChIP)

ChIP analysis was performed as previously described^43^. Briefly, cells with 2 μM DOX treatment were cross-linked in 1% formaldehyde, and the DNA was sonicated into a size range of 200–1000 base pairs using a Bioruptor Sonicator (Diagenode) for five cycles of 3 son/3 s off. The extracts were pre-cleared in BSA-blocked protein A/G beads and incubated with antibody against H3K27ac (#8173, dilution 1:100, Cell Signaling Technology, Inc) or control IgG (#2729, dilution 1:100, Cell Signaling Technology, Inc) overnight at 4 °C. After being washed, the DNA was eluted and reverse-cross-linked overnight at 65 °C. Purified ChIP DNA was confirmed by Quantitative RT-PCR. PFKM primers sequences: 5′-CAACACCACCACTACCTT-3′ (forward), 5′-CACTGCCATCAAACAAAC-3′ (reverse).

### Statistical analysis

Data were expressed as mean ± SD and analyzed by Prism8.4.2. Comparisons between 2 groups were performed with Student's *t* test, and multiple comparisons were performed with one-way ANOVA. *P* < 0.05 was defined statistically significant.

## Supplementary Information

Supplementary Figure S1.

Supplementary Figure S2.

Supplementary Figure S3.

Supplementary Figure S4.

Supplementary Figure S5.

Supplementary Figure S6.

Supplementary Figure S7.

Supplementary Figure S8.

Supplementary Figure S9.

Supplementary Figure S10.

Supplementary Figure S11.

Supplementary Figure S12.

Supplementary Figure S13.

Supplementary Figure S14.

Supplementary Figure S15.

Supplementary Figure S16.

Supplementary Figure S17.

Supplementary Figure S18.

Supplementary Information 1.

## Data Availability

We confirm that all data generated or analyzed during this study are available from the corresponding author on reasonable request.
