# Supplementary figures and images for: PFKM inhibits doxorubicin-induced cardiotoxicity by enhancing oxidative phosphorylation and glycolysis
Source: Sci Rep. 2022 Jul 8;12:11684. doi: 10.1038/s41598-022-15743-0 (PMC9266090; doi:10.1038/s41598-022-15743-0)

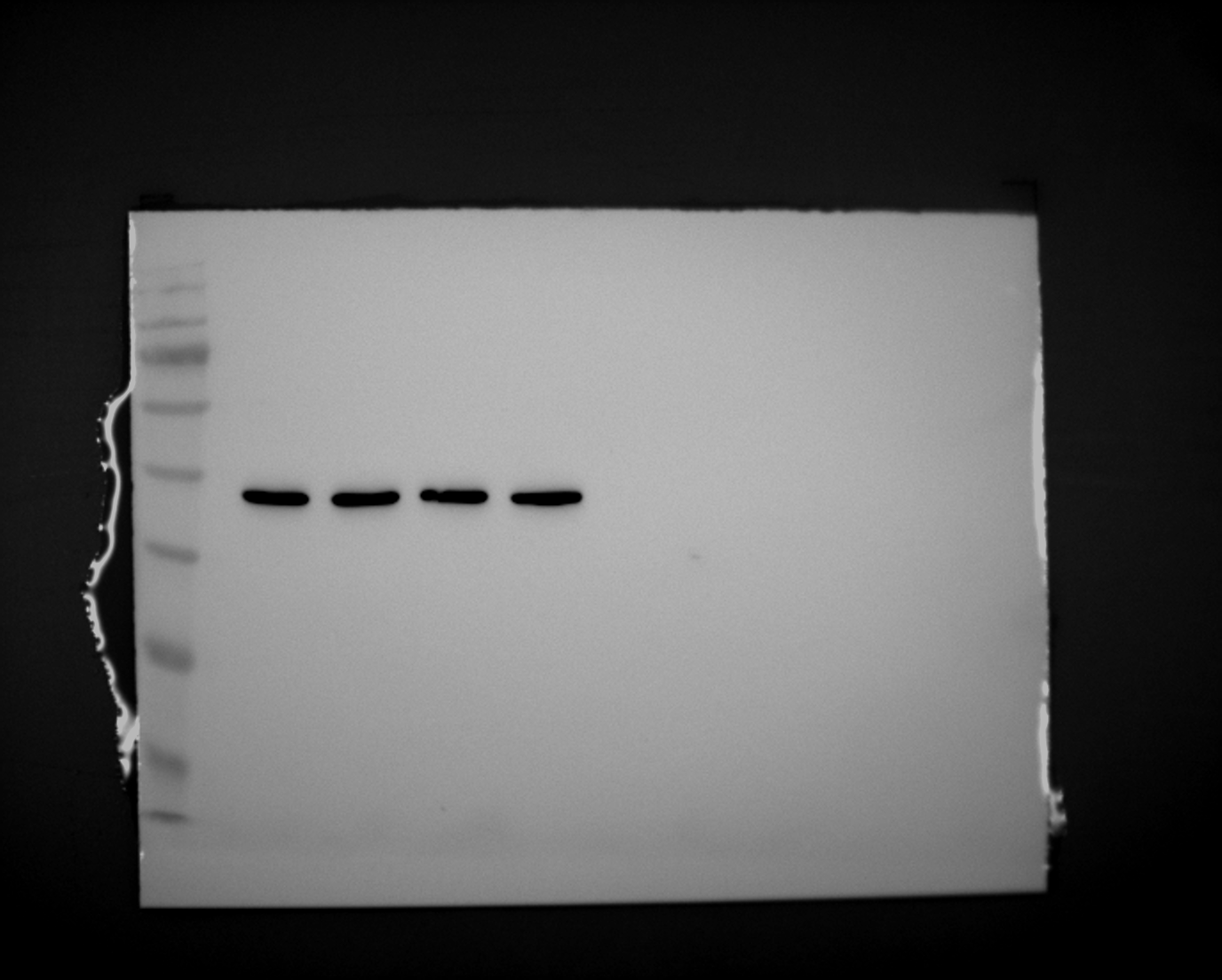

Supplement: Supplementary file 1 — Supplementary Figure S1. [file 41598_2022_15743_MOESM1_ESM.tif]

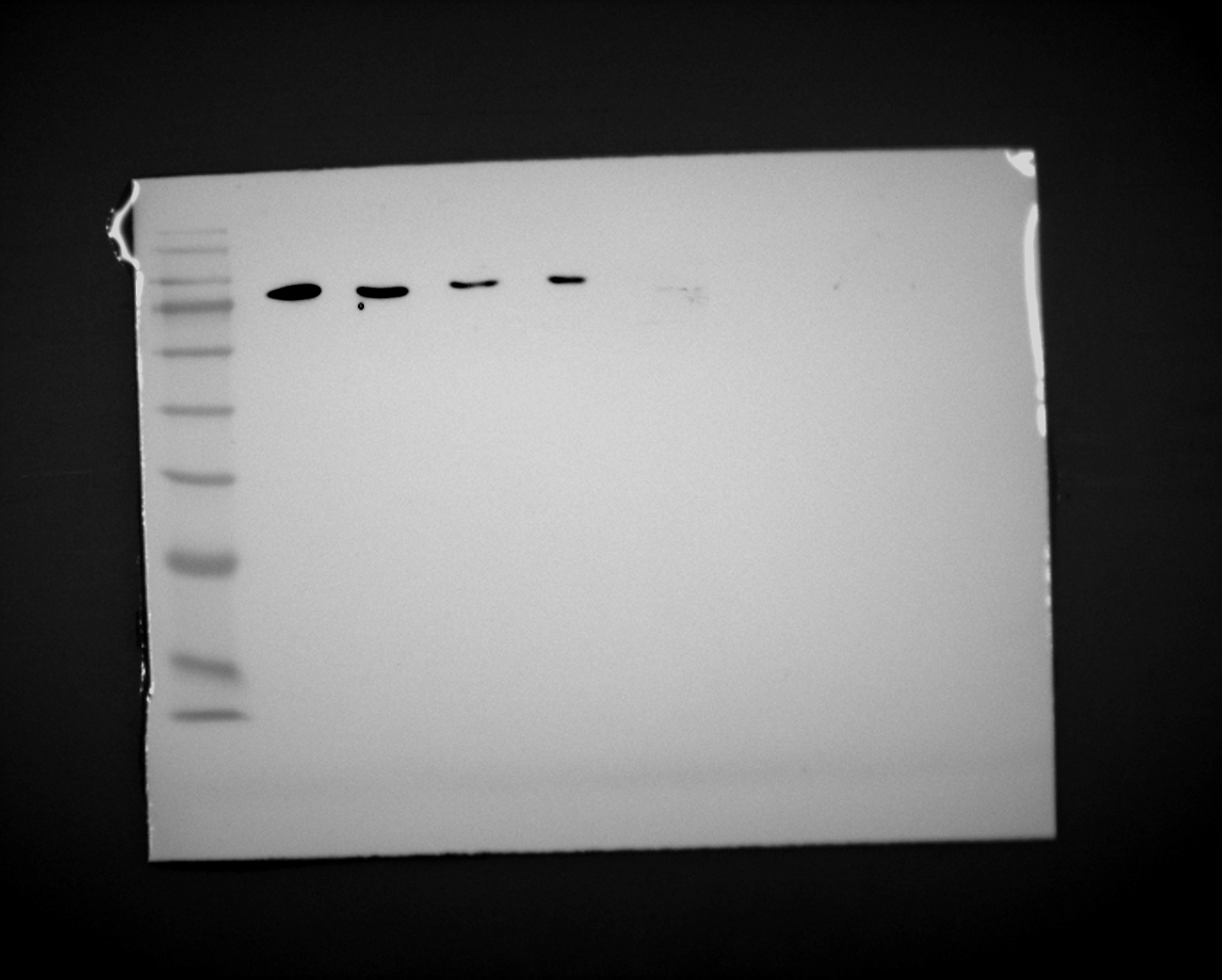

Supplement: Supplementary file 2 — Supplementary Figure S2. [file 41598_2022_15743_MOESM2_ESM.tif]

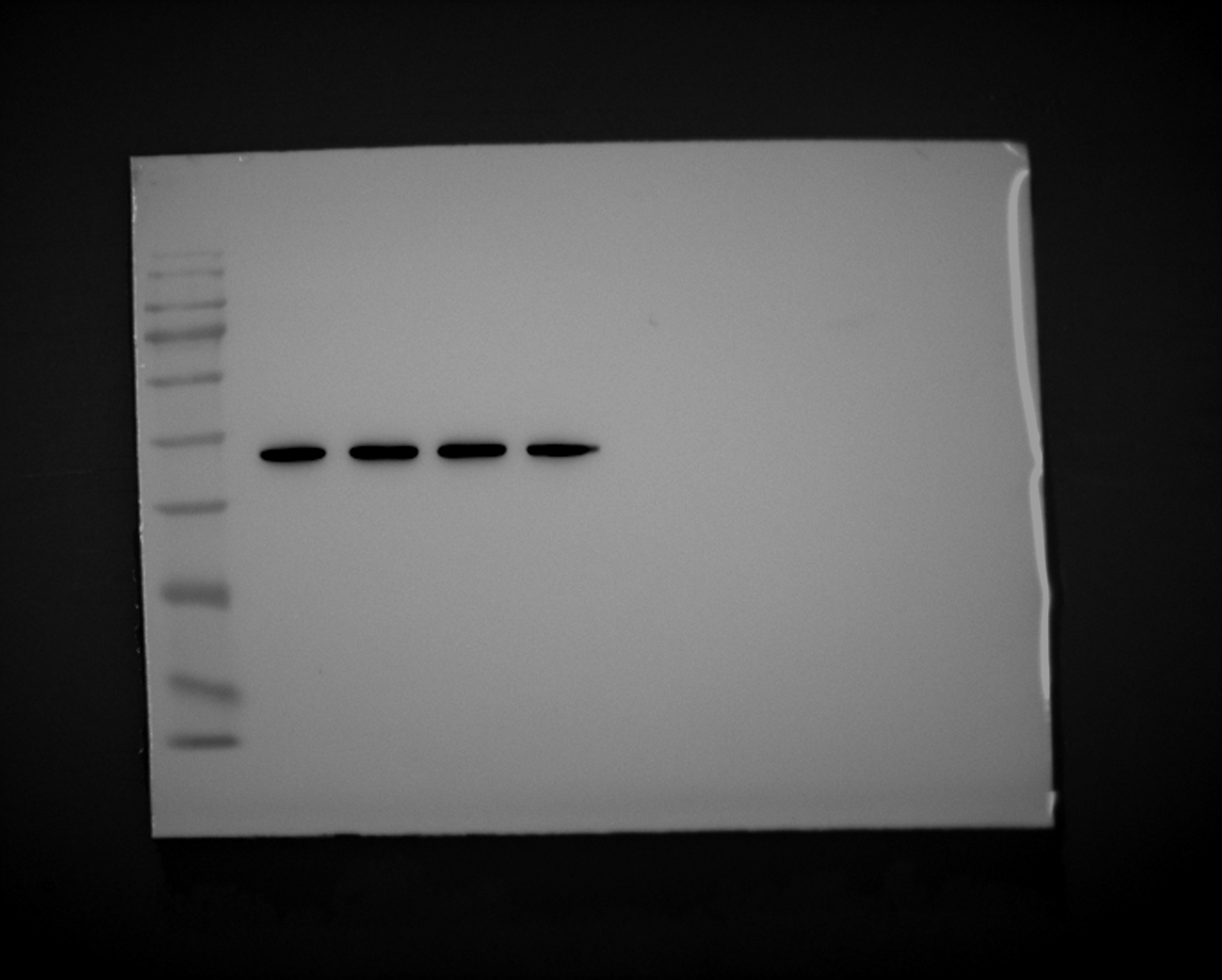

Supplement: Supplementary file 3 — Supplementary Figure S3. [file 41598_2022_15743_MOESM3_ESM.tif]

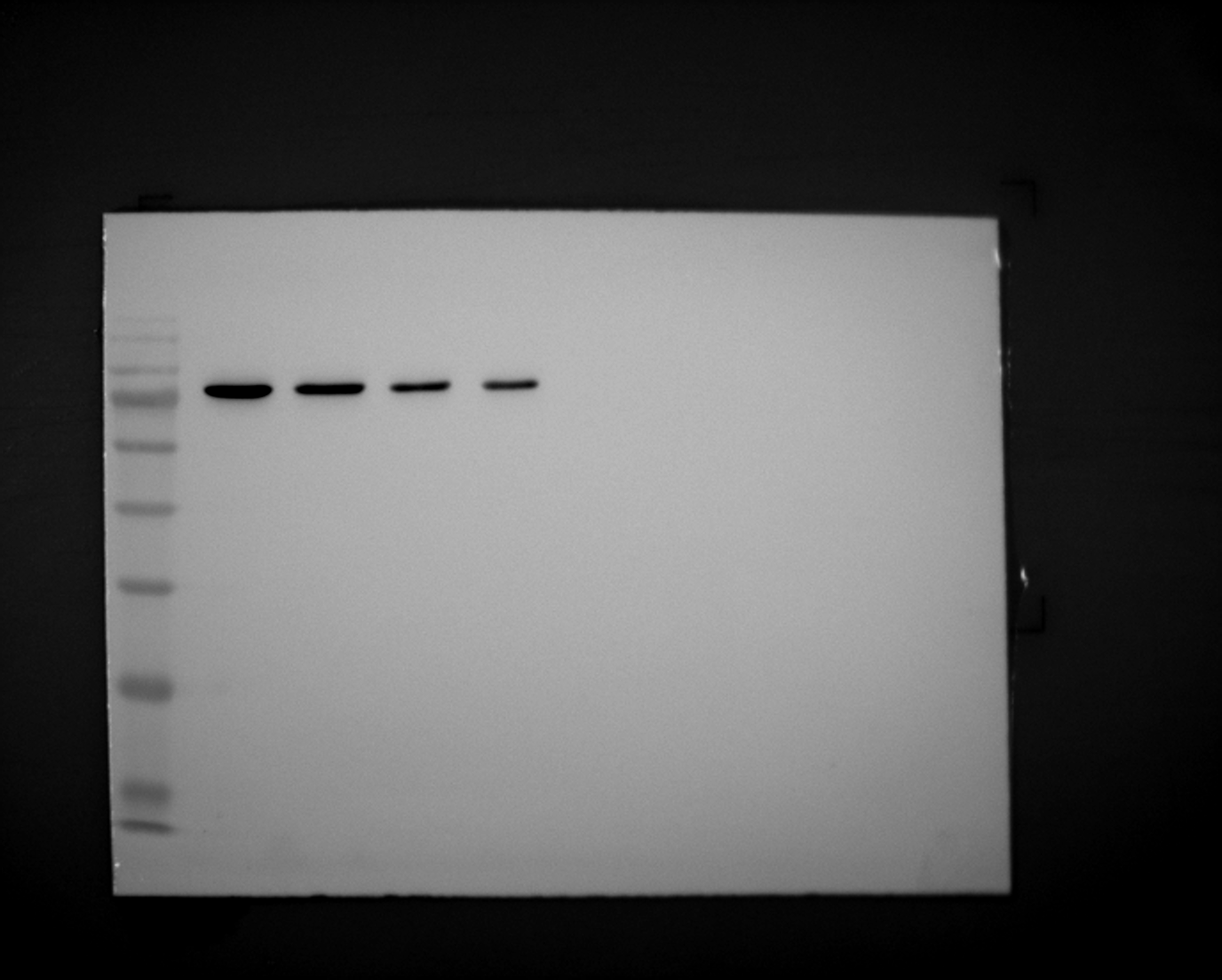

Supplement: Supplementary file 4 — Supplementary Figure S4. [file 41598_2022_15743_MOESM4_ESM.tif]

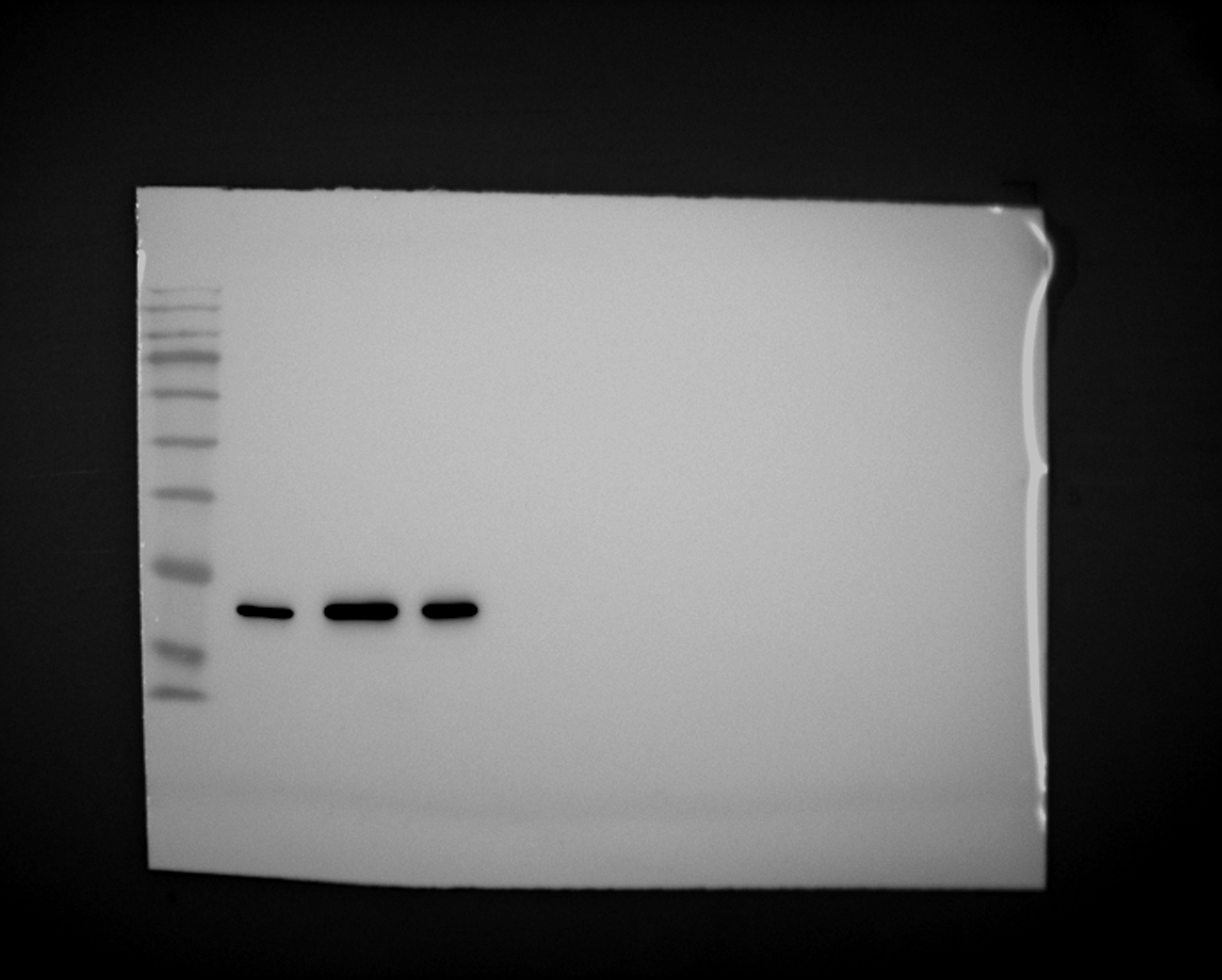

Supplement: Supplementary file 5 — Supplementary Figure S5. [file 41598_2022_15743_MOESM5_ESM.tif]

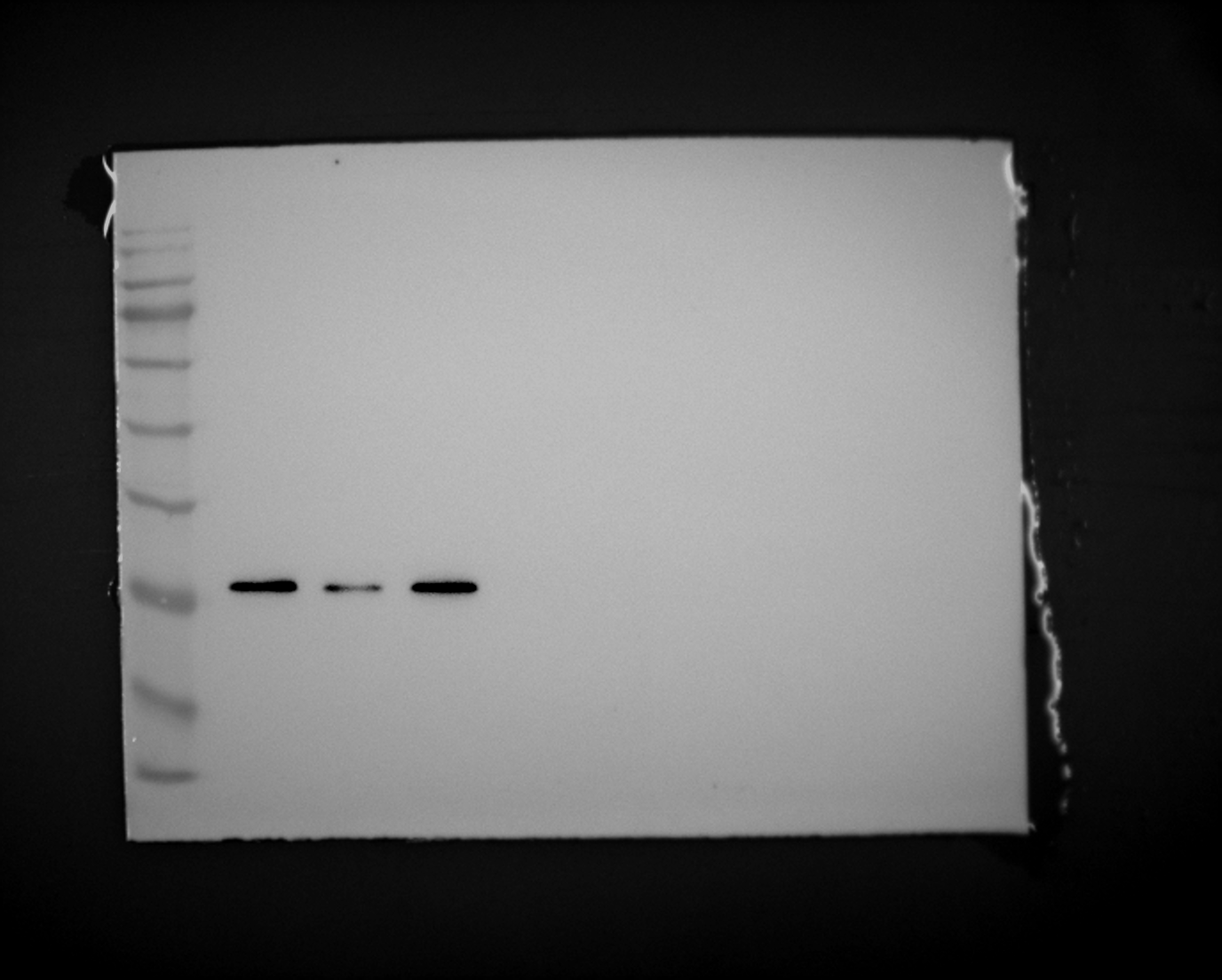

Supplement: Supplementary file 6 — Supplementary Figure S6. [file 41598_2022_15743_MOESM6_ESM.tif]

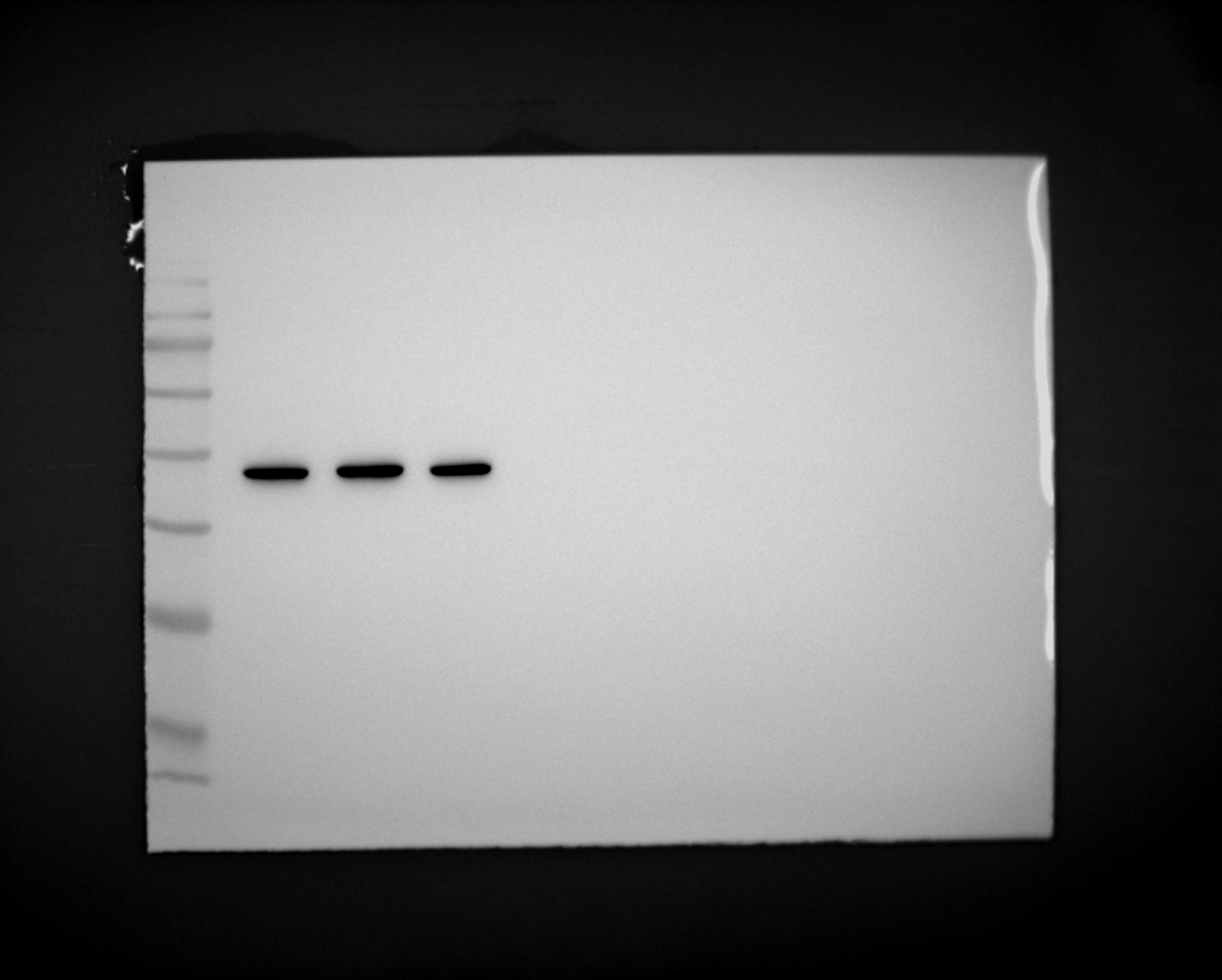

Supplement: Supplementary file 7 — Supplementary Figure S7. [file 41598_2022_15743_MOESM7_ESM.tif]

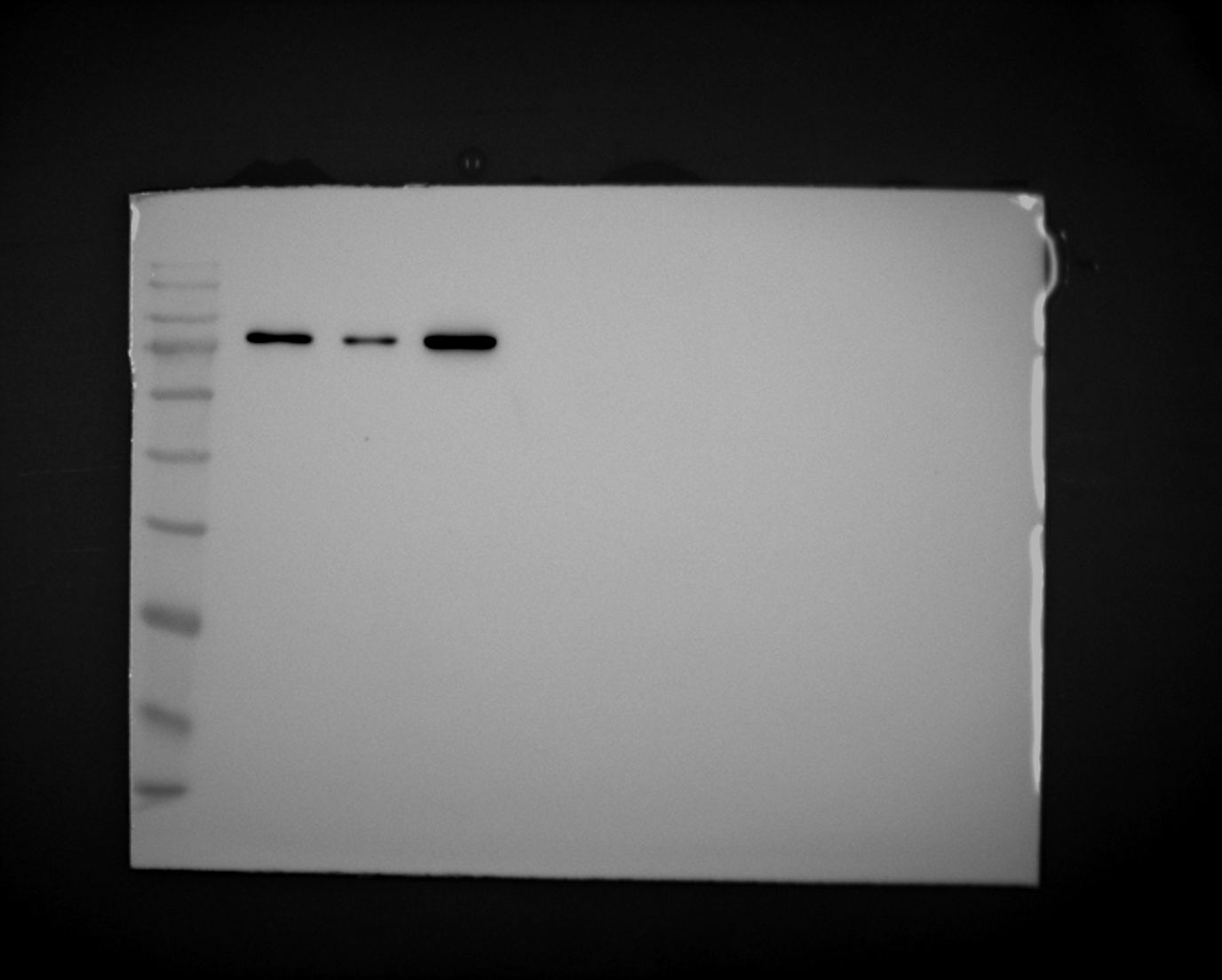

Supplement: Supplementary file 8 — Supplementary Figure S8. [file 41598_2022_15743_MOESM8_ESM.tif]

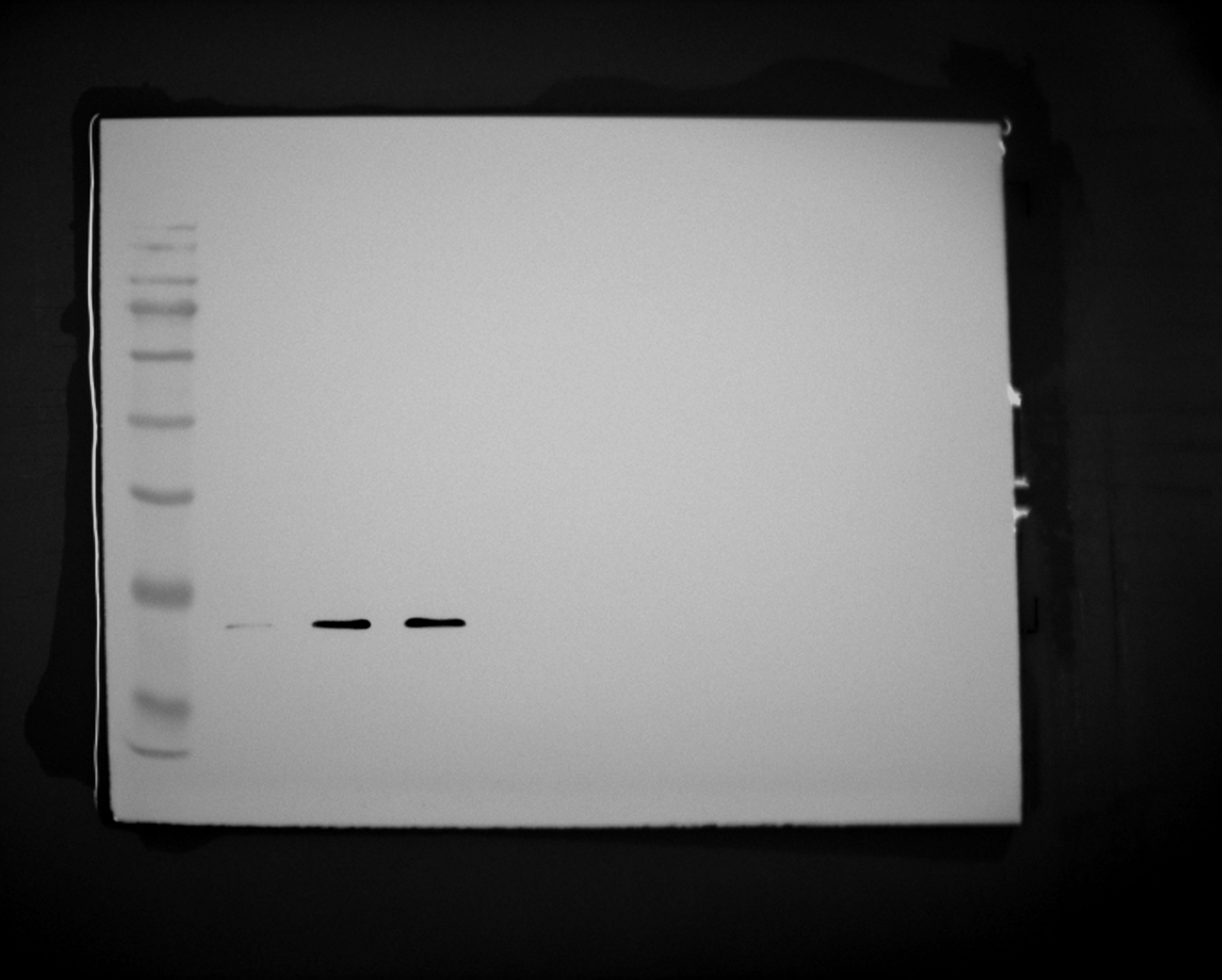

Supplement: Supplementary file 9 — Supplementary Figure S9. [file 41598_2022_15743_MOESM9_ESM.tif]

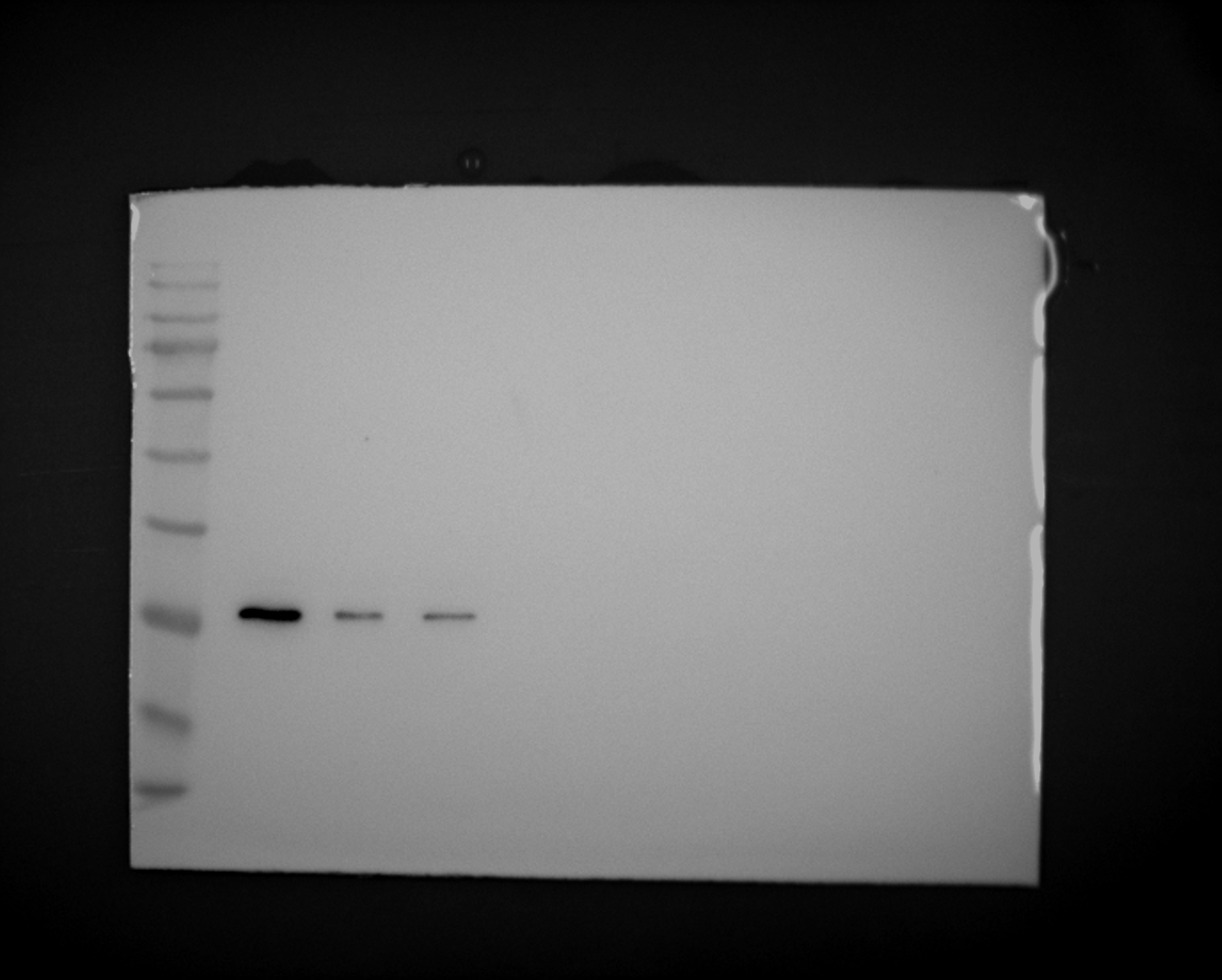

Supplement: Supplementary file 10 — Supplementary Figure S10. [file 41598_2022_15743_MOESM10_ESM.tif]

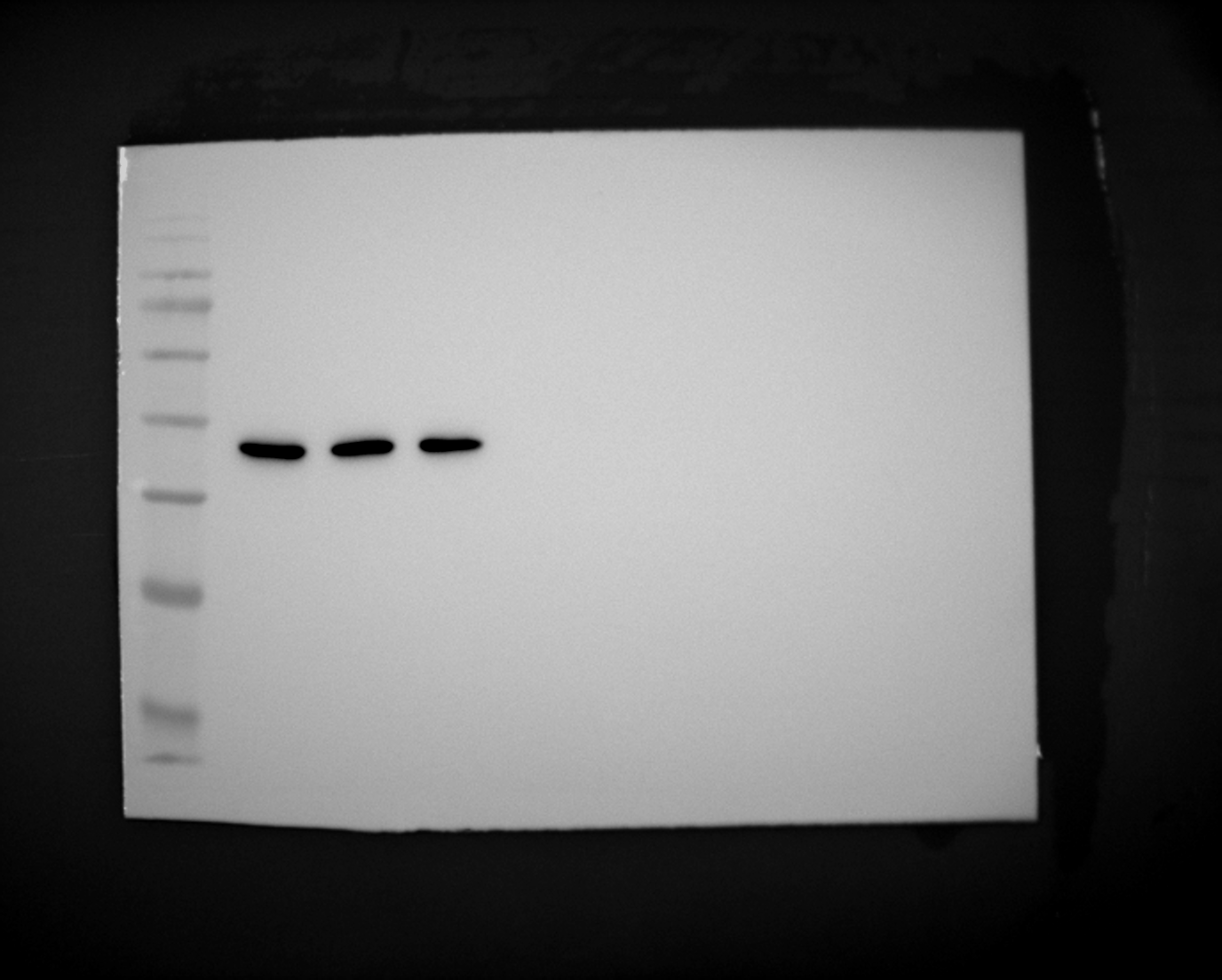

Supplement: Supplementary file 11 — Supplementary Figure S11. [file 41598_2022_15743_MOESM11_ESM.tif]

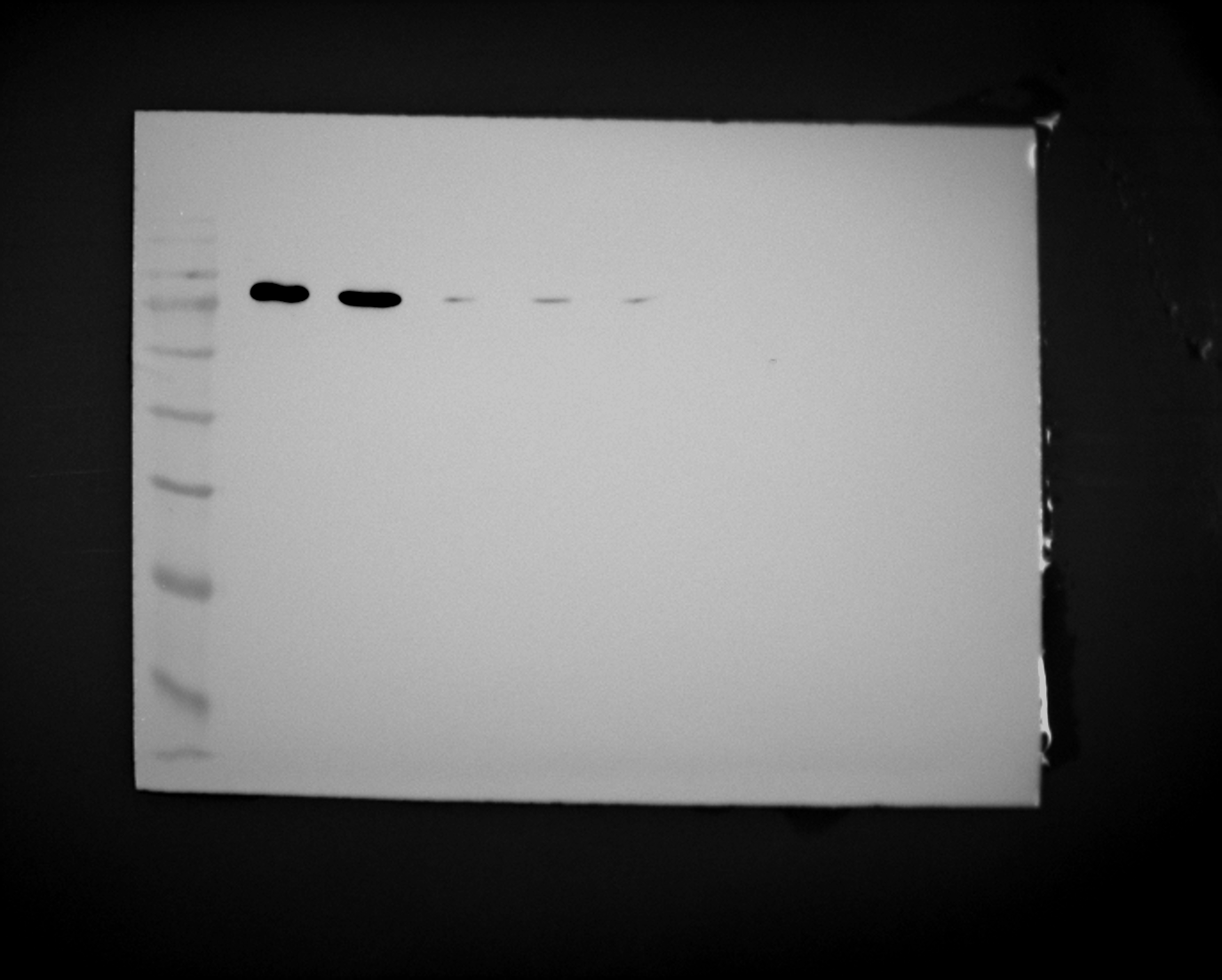

Supplement: Supplementary file 12 — Supplementary Figure S12. [file 41598_2022_15743_MOESM12_ESM.tif]

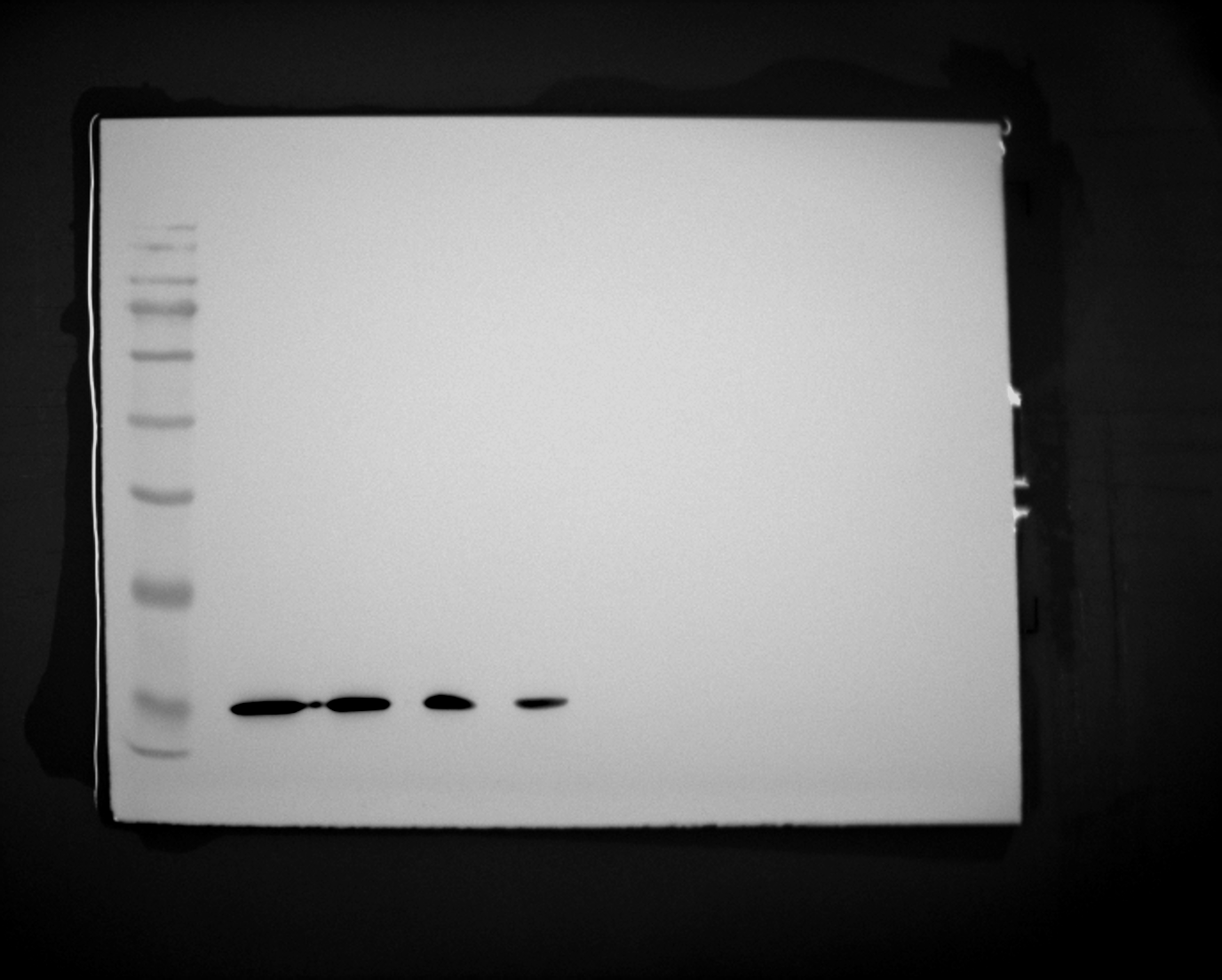

Supplement: Supplementary file 13 — Supplementary Figure S13. [file 41598_2022_15743_MOESM13_ESM.tif]

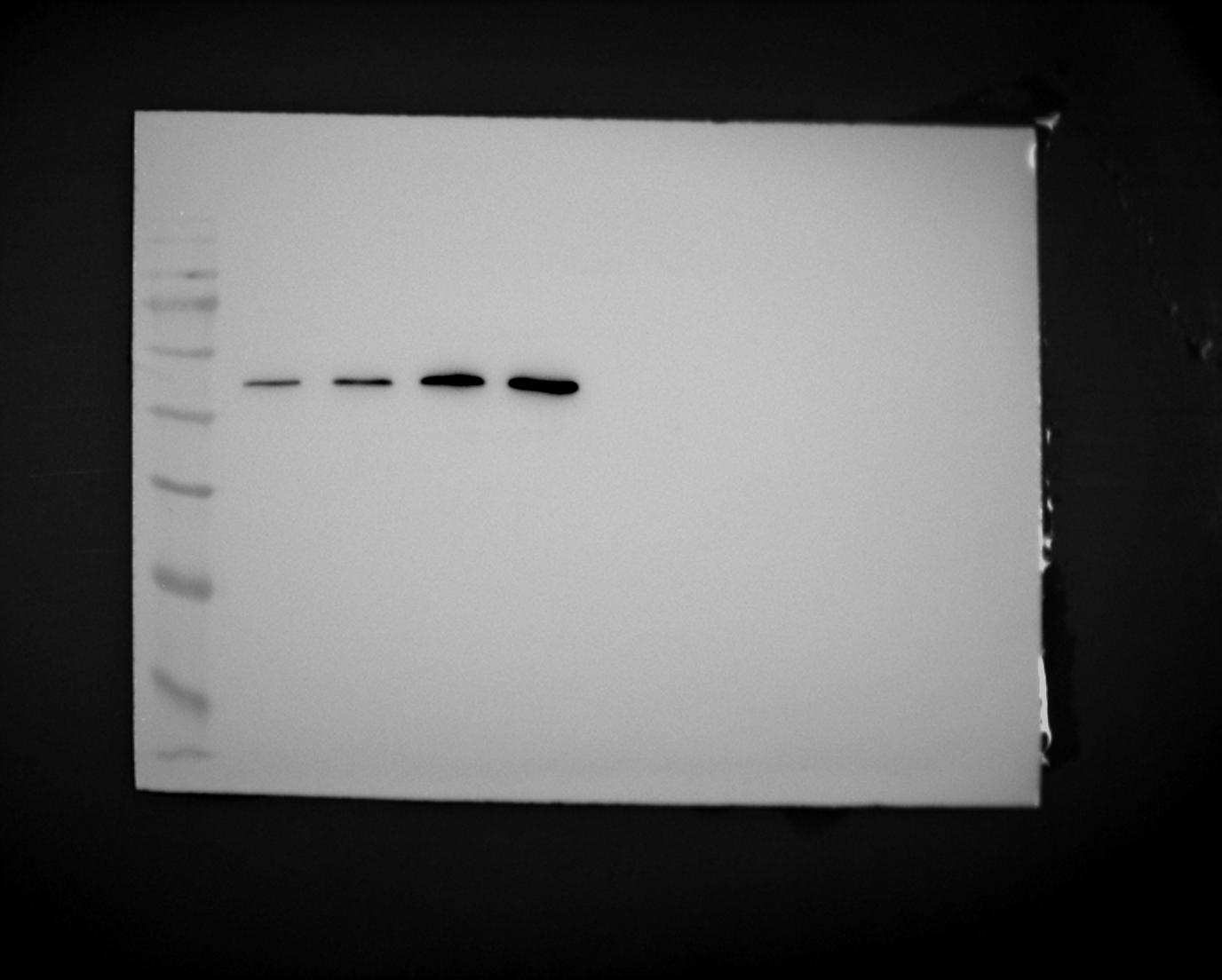

Supplement: Supplementary file 14 — Supplementary Figure S14. [file 41598_2022_15743_MOESM14_ESM.tif]

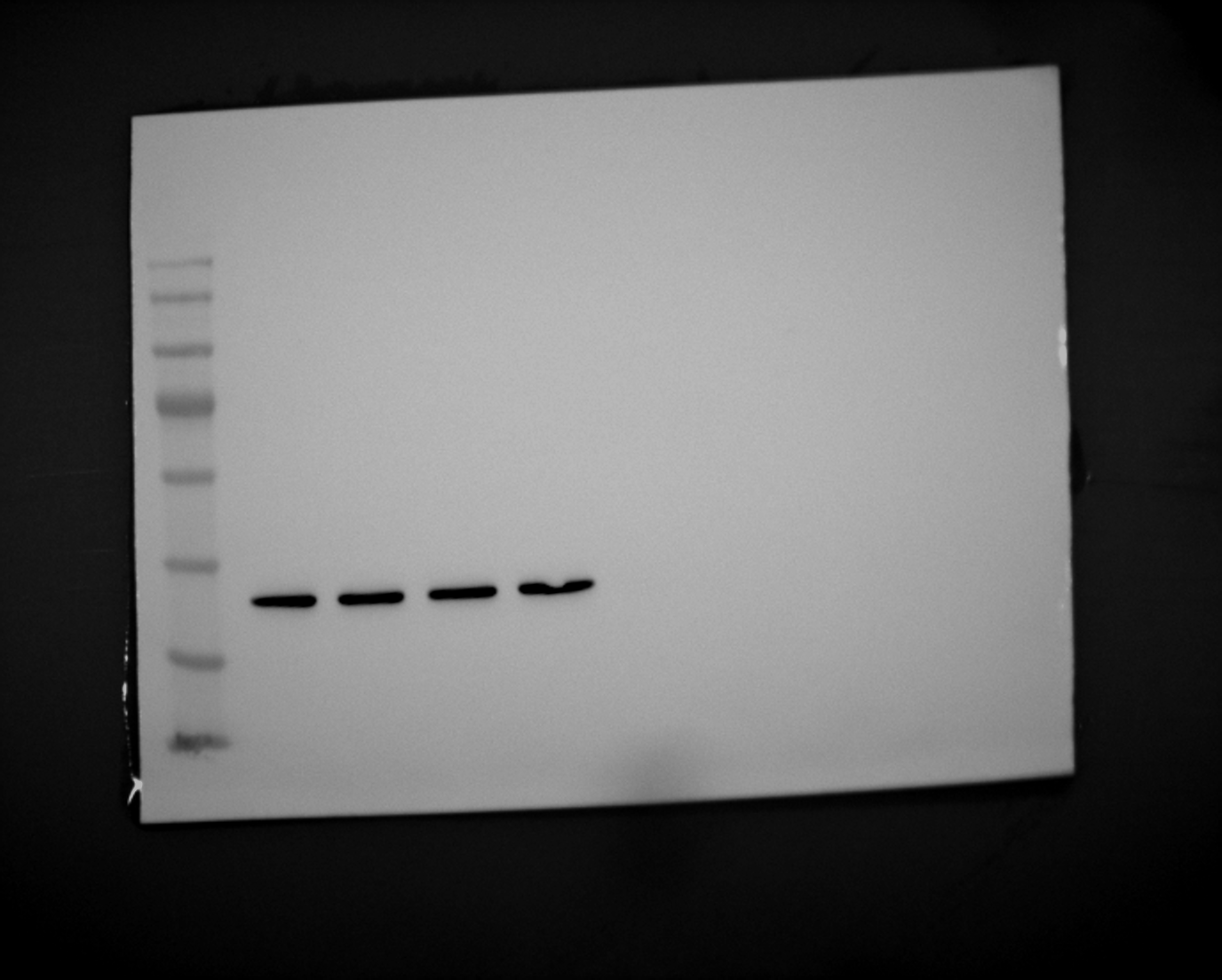

Supplement: Supplementary file 15 — Supplementary Figure S15. [file 41598_2022_15743_MOESM15_ESM.tif]

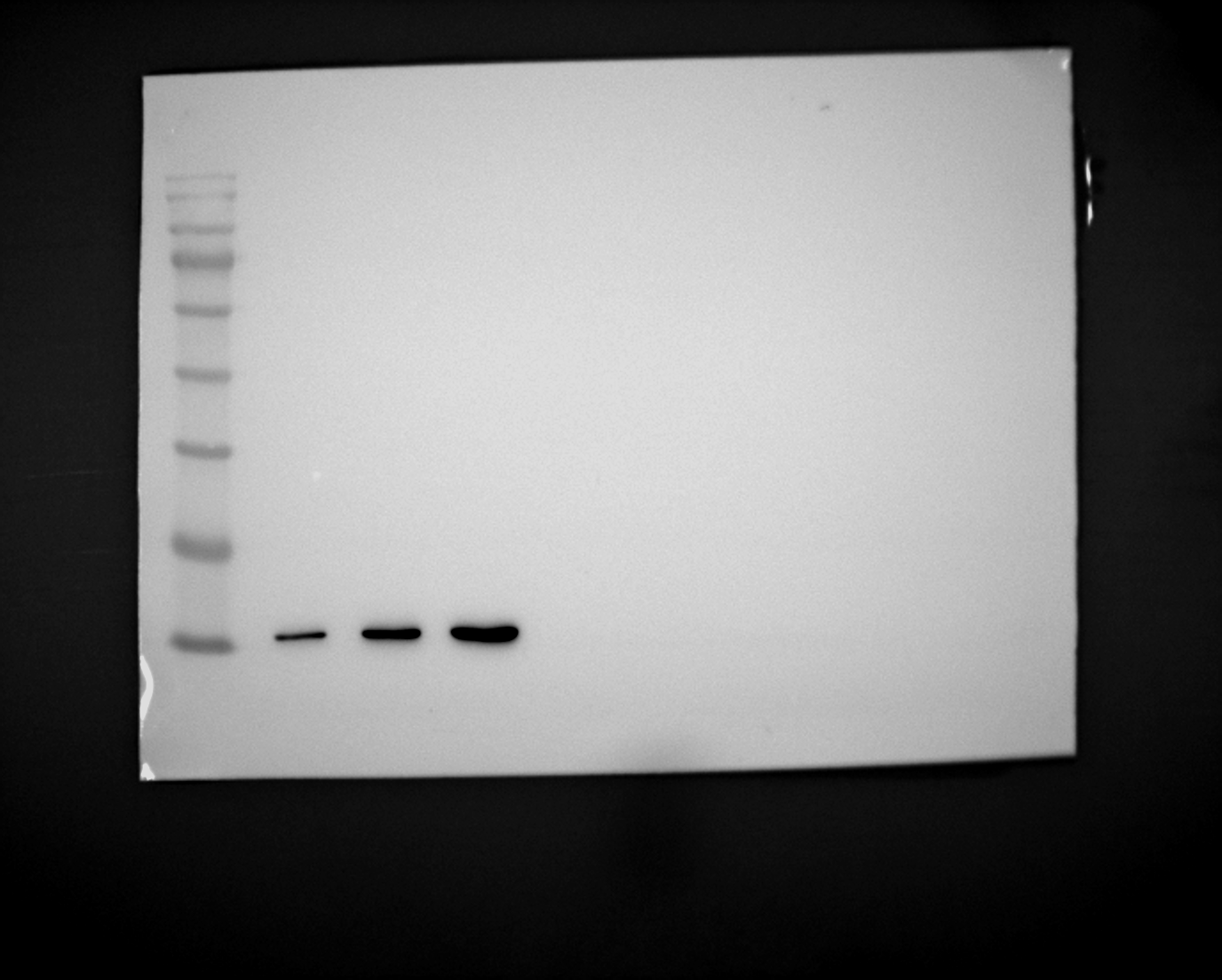

Supplement: Supplementary file 16 — Supplementary Figure S16. [file 41598_2022_15743_MOESM16_ESM.tif]

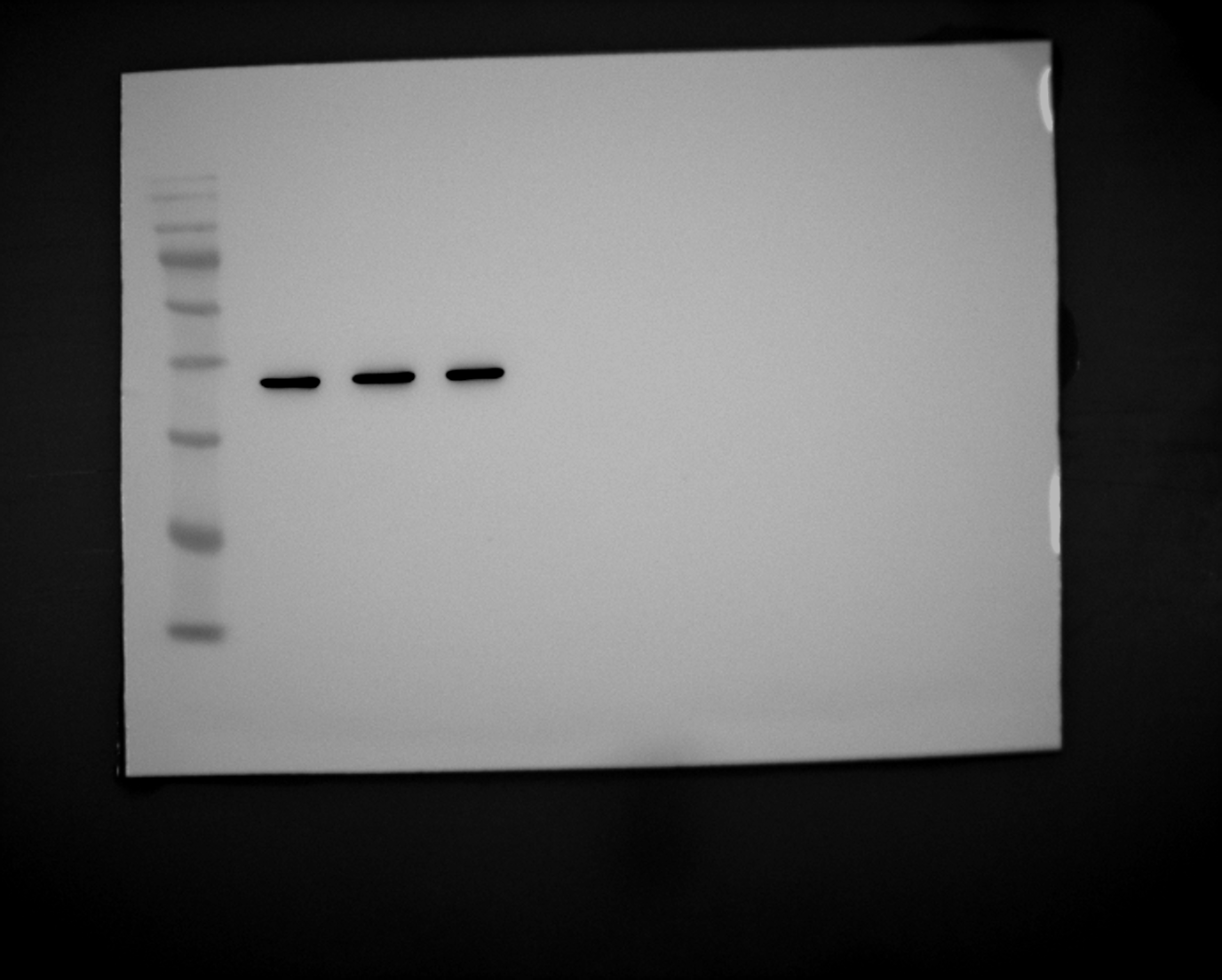

Supplement: Supplementary file 17 — Supplementary Figure S17. [file 41598_2022_15743_MOESM17_ESM.tif]

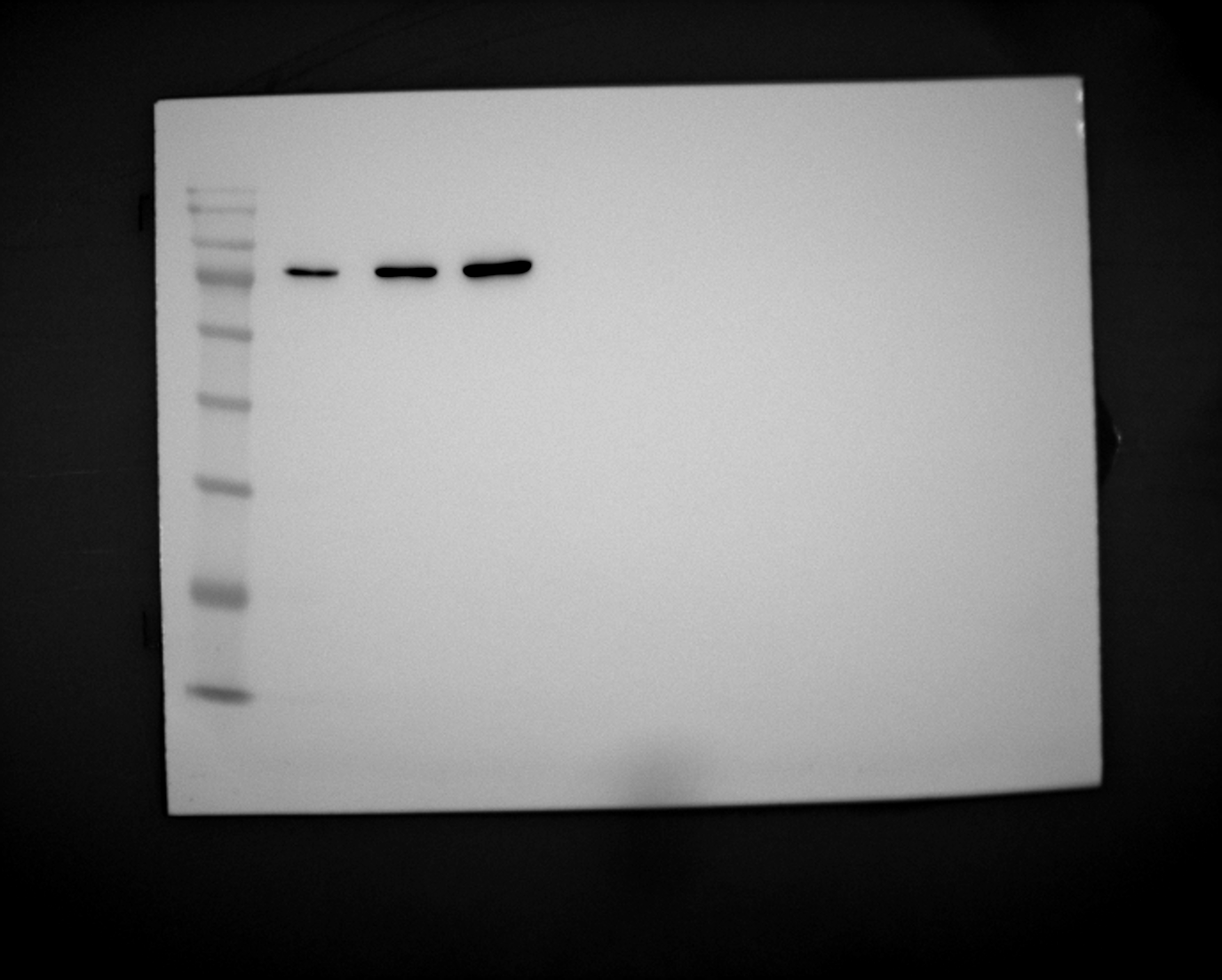

Supplement: Supplementary file 18 — Supplementary Figure S18. [file 41598_2022_15743_MOESM18_ESM.tif]
