# Supplementary material for: PFKM inhibits doxorubicin-induced cardiotoxicity by enhancing oxidative phosphorylation and glycolysis
Source: Sci Rep. 2022 Jul 8;12:11684. doi: 10.1038/s41598-022-15743-0 (PMC9266090; doi:10.1038/s41598-022-15743-0)
Supplement: Supplementary file 19 — Supplementary Information 1. [file 41598_2022_15743_MOESM19_ESM.docx]

The grouping order: Control, si-Negative Control (si-NC), siRNA-1 (site 1), siRNA-2 (site 2) and siRNA-3 (site 3)，we cut the original image and only kept si-NC, siRNA-1 and siRNA-2 in the Figure-3D.
